# Mapping the missing: a scoping review identifying critically underrepresented LGBTQI+ youth within online sexual, reproductive, and transgender healthcare research

**DOI:** 10.1080/26410397.2026.2679359

**Published:** 2026-05-29

**Authors:** Julie McLeod, Claudia S. Estcourt, Paul Flowers, Jo Gibbs, Jennifer MacDonald

**Affiliations:** a PhD Candidate (Psychology), Glasgow Caledonian University (Sexual Health and BBV Research Group), Glasgow, Scotland.; b Professor of Sexual Health and HIV, Glasgow Caledonian University (Sexual Health and BBV Research Group), Glasgow, Scotland; c Professor of Health Change, University of Strathclyde, Glasgow, Scotland; d Principal Clinical Research Fellow, University College London (Institute for Global Health), London, England; e Research Fellow, Glasgow Caledonian University (Sexual Health and BBV Research Group), Glasgow, Scotland

**Keywords:** LGBT, sexual minority, gender minority, digital health, eHealth, mHealth, inequalities, gender affirming care, health equity, sexual health care

## Abstract

Online sexual, reproductive, and transgender healthcare can overcome barriers to care among lesbian, gay, bisexual, trans, queer/questioning, intersex, and other (LGBTQI+) youth and address disproportionately poor sexual and reproductive health outcomes. However, LGBTQI+ youth are heterogenous and online healthcare spans broad health topics and online platforms. To map recent research and identify gaps, we conducted a scoping review, following Joanna Briggs Institute methodology, using the Participants (LGBTQI+ youth aged 10–35 years), Concept (online sexual, reproductive, and transgender healthcare), Context (high-income countries) eligibility framework. We searched nine databases for recent literature (2018–2024), two reviewers screened studies using Rayyan, and data were extracted to Excel and analysed descriptively (*N* = 132 included papers). Most papers (89/132) were from distinct studies; 43/132 were from 15 studies. There were quantitative (57/132), qualitative (41/132), and mixed methods studies (34/132). Most focused on sexual healthcare (95/132) including HIV/STI prevention (68/95) and HIV management (10/95); 30/132 on transgender healthcare; and only 3/132 on reproductive healthcare. Most targeted young men who have sex with men (79/132) or trans and gender-diverse youth (44/132). Only 4/132 targeted young sexual minority women. Almost all were from the US (119/132). Amid a global shift to delivering healthcare online, this timely review provides the first comprehensive map of critical blind spots, highlighting the urgency of research on reproductive health, sexual wellbeing, and sexual minority women. Addressing these gaps is essential for providing equitable healthcare and reducing health disparities. These findings can guide the delivery of online healthcare that meets the needs of all LGBTQI+ youth.

## Plain language summary

Young people who are lesbian, gay, bisexual, trans, queer, intersex, or part of the LGBTQI+ community can struggle to get in-person sexual and reproductive healthcare. This can be because of worries about privacy or judgment. Online healthcare can make it easier for LGBTQI+ youth to get sexual and reproductive care. Healthcare is moving online but it is usually made for people who are not LGBTQI+. This means that online healthcare may not meet the needs of LGBTQI+ youth. So, we wanted to know what online healthcare research exists for LGBTQI+ youth and who is being left out. To do this, we looked at 132 published studies from 2018 to 2024. These studies explored online sexual, reproductive, and transgender healthcare for LGBTQI+ youth (aged 10 and 35). We included transgender healthcare because this can often involve reproductive and fertility care. We only looked at studies from places like the United States, United Kingdom, Australia, Canada, and Europe. We found that most of the studies were about sexual health for young gay and bisexual men. Very few studies looked at reproductive health, sexual wellbeing, or sexual violence. Very few studies included young lesbian or bisexual women or trans youth. Almost all studies came from the United States. Our findings are the first to show that it is urgent for more research to focus on young sexual minority women and trans youth. This is the only way to make sure that online sexual and reproductive healthcare meets the needs of all LGBTQI+ youth.

## Résumé

Les services de santé sexuelle et reproductive (SSR) en ligne et les soins de santé pour les personnes transgenres peuvent lever les obstacles à l’accès aux soins pour les jeunes lesbiens, gays, bisexuels, transgenres, queers/en questionnement, intersexes et autres (LGBTQI+) et améliorer leurs résultats anormalement mauvais en matière de SSR. Néanmoins, les jeunes LGBTQI+ forment un groupe hétérogène et les services de SSR couvrent un large éventail de thèmes de santé et de plateformes en ligne. Par conséquent, afin de recenser les publications récentes et d’identifier les lacunes de la recherche, nous avons mené une étude de la portée, suivant la méthodologie du Joanna Briggs Institute, au moyen du cadre d’éligibilité suivant: participants (jeunes LGBTQI+ âgés de 10 à 35 ans), concept (services de SSR et soins de santé pour personnes transgenres) et contexte (pays à revenu élevé). Neuf bases de données ont été consultées pour des publications parues entre 2018 et 2024. Deux examinateurs ont sélectionné les études indépendamment à l’aide de l’outil Rayyan. Les données des 132 études incluses ont été extraites vers Excel et analysées de manière descriptive. La plupart des articles (89/132) provenaient d’études distinctes, alors que 43/132 étaient tirés de 15 études. On a dénombré des études quantitatives (57/132), qualitatives (41/132) et à méthode mixte (34/132). La plupart se concentraient sur la santé sexuelle (95/132), notamment la prévention du VIH et des IST (68/95) et la prise en charge du VIH (10/95), ainsi que sur la santé des personnes transgenres (30/132). Trois seulement abordaient la santé reproductive. La majorité des études ciblaient les jeunes hommes ayant des rapports sexuels avec des hommes (79/132) ou des jeunes transgenres et de genre divers (44/132). Seules quatre études sur 132 visaient les jeunes femmes appartenant à des minorités sexuelles. Presque toutes provenaient des États-Unis (119/132). Dans un contexte de réorientation mondiale en faveur de la prestation de soins de SSR en ligne, cet examen utile est le premier à recenser complètement les lacunes les plus criantes, soulignant l’urgence de la recherche sur la santé reproductive, le bien-être sexuel et les femmes appartenant à des minorités sexuelles. Combler ces lacunes est essentiel pour assurer des soins de SSR équitables et réduire les inégalités de santé. Ces résultats peuvent guider les professionnels de santé, les décideurs politiques et les concepteurs de plateformes de santé numérique afin de garantir des soins de SSR qui répondent aux besoins de tous les jeunes LGBTQI+.

## Resumen

Los servicios de salud sexual y reproductiva (SSR) y servicios de salud transgénero en línea pueden superar las barreras que enfrentan las personas lesbianas, gay, bisexuales, trans, queer/cuestionándose, intersexuales y otras personas jóvenes (LGBTQI+) para acceder a los servicios de salud y abordar resultados de salud sexual y reproductiva desproporcionadamente deficientes. Sin embargo, las personas jóvenes LGBTQI+ son heterogéneas y los servicios de SSR en línea abarcan amplios temas de salud y plataformas digitales. Por consiguiente, para mapear la literatura reciente e identificar brechas de investigación, realizamos una revisión de alcance, siguiendo la metodología del Instituto Joanna Briggs, utilizando el marco de elegibilidad del Concepto (servicios de SSR y servicios de salud transgénero en línea) y Contexto (países de altos ingresos) de Participantes (personas jóvenes LGBTQI+ de 10 a 35 años). En nueve bases de datos, se realizó una búsqueda bibliográfica de estudios publicados entre 2018 y 2024. Dos revisores examinaron los estudios independientemente utilizando Rayyan. Los datos de 132 estudios incluidos fueron extraídos a Excel y analizados de manera descriptiva. La mayoría de los artículos (89/132) eran de diferentes estudios; 43/132 eran de uno de quince estudios. Había estudios cuantitativos (57/132), cualitativos (41/132) y de métodos mixtos (34/132). La mayoría se centraba en servicios de salud sexual (95/132), tales como prevención de VIH/ITS (68/95) y manejo del VIH (10/95) y servicios de salud transgénero (30/132). Solo tres estudios abordaban servicios de salud reproductiva. La mayoría de los estudios se enfocaban en hombres jóvenes que tienen sexo con hombres (79/132) o en jóvenes trans y de género diverso (44/132). Solo 4/132 se centraban en mujeres jóvenes de minorías sexuales. Casi todos eran de Estados Unidos (119/132). En medio de la transición mundial hacia la prestación de servicios de SSR en línea, esta revisión oportuna ofrece el primer mapa integral de puntos ciegos críticos y destaca la urgencia de realizar investigaciones sobre salud reproductiva, bienestar sexual y mujeres de minorías sexuales. Abordar estas brechas es esencial para ofrecer servicios de SSR equitativos y reducir las disparidades de salud. Estos hallazgos pueden guiar a profesionales de salud, responsables políticos y diseñadores de servicios de salud digitales en la prestación de servicios de SSR que atiendan las necesidades de todas las personas jóvenes LGBTQI+.

## Introduction

Lesbian, gay, bisexual, trans, queer, questioning, intersex, and other sexual orientation and gender-diverse (LGBTQI+) youth face a disproportionate burden of poor sexual and reproductive health, including high rates of sexually transmitted infections (STIs) and blood borne viruses (BBVs), sexual violence and abuse, low sexual wellbeing, and unplanned pregnancy at a young age (see Supplementary File 1 for key terms used throughout this paper). In particular, young gay, bisexual and other men and people assigned male at birth who have sex with men (GBMSM) and trans women who have sex with men are at higher risk for STIs and BBVs.^1–3^ Additionally, young bisexual women and trans youth are at higher risk for unplanned pregnancy and sexual violence and abuse than their cisgender and heterosexual peers.^3–5^

Despite these risks, LGBTQI+ youth have low uptake of sexual and reproductive health (SRH) care, such as STIs/BBVs testing, human papilloma virus vaccination, and uptake of pre-exposure prophylaxis (PrEP).^6–8^ Multiple barriers contribute to this issue, including confidentiality concerns, lack of perceived risk or necessity, discrimination and stigma, healthcare providers’ lack of knowledge and training about their needs, and cisnormative and heteronormative assumptions of their gender and sexuality.^6–10^

Moreover, for trans and gender-diverse (TGD) youth, a critical issue relating to sexual and reproductive health is transgender health.^11^ Transgender health is the ability for TGD individuals to live in the gender that feels most authentic and comfortable.^12^ This is based on the premise that gender and sex are distinct^12^ and that some individuals experience emotional distress, known as gender dysphoria, due to incongruence between their gender and sex.^13^ Gender dysphoria and related anxiety and depression can reduce uptake of SRH care.^14,15^ Conversely, receiving gender-affirming care, such as hormones and/or surgery,^16^ is associated with increased engagement with SRH care among TGD youth, including STI testing.^9^ Broader transgender healthcare, which encompasses information, non-clinical support, and clinical care regarding gender identity, expression, and transition,^17^ is also intrinsically linked with reproductive healthcare, such as fertility preservation or assistance.^18^ Therefore, it is critical to ensure that transgender health is considered appropriately alongside sexual and reproductive health.

In addition to gender and sexuality, other intersecting social factors associated with increased risk of poor health outcomes, such as race/ethnicity and socio-economic status,^19,20^ can shape LGBTQI+ youth's sexual and reproductive health outcomes and experience of SRH care. In the United Kingdom (UK) and United States of America (US), for instance, proportionately, young Black GBMSM bear the largest burden of HIV incidence.^2, 21^ Further, young LGBTQI+ people of colour in the US and Canada have reported that stigma influences their decision to disclose their sexual identity to healthcare providers and/or to consider uptake of PrE.^22^

Online SRH care, such as information,^23^ non-clinical support (e.g. live chat with a healthcare professional^24^), and clinical care (e.g. online postal STI testing^25^) has the potential to overcome barriers and increase access to care for LGBQTI+ youth by offering privacy, convenience, accessibility, and reduced stigma.^26, 27^ LGBTQI+ youth are more likely to seek sexual health information online than from other sources, such as school, healthcare professionals, or family.^28, 29^ Importantly, the delivery of online SRH care has grown over the past five years, particularly in the UK and other high-income countries,^30–32^ expedited by the COVID-19 pandemic.^33^ However, research into online sexual and reproductive health innovations has typically focussed on general populations,^25,34–37^ risking online services not meeting the needs of LGBTQI+ youth. This has been indicated by a systematic review which found that LGBTQI+ youth have reported issues with online SRH care being heteronormative and cisnormative.^38^

Given the potential for online SRH care to increase access and reduce health disparities among LGBTQI+ youth, it is essential to understand how online services are currently designed and delivered for this population. However, online SRH care spans a wide range of health areas (e.g. fertility,^39^ wellbeing,^40^ violence/abuse,^41^ infection^42^), healthcare types (e.g. information,^23^ non-clinical support,^24^ clinical care^25^), and online formats (e.g. websites,^23^ mobile apps,^40^ SMS test messaging^39^). Thus, the breadth and nature of research into online SRH care for LGBTQI+ youth is unclear. A review is needed to map recent literature to identify where there are gaps^43^ in order to guide future research and service development.

Finally, when seeking to improve the sexual health of LGBTQI+ youth, it is critical to explore how theory, models, and frameworks (henceforth theory) are used in research. For intervention development and evaluation, the use of behavioural and implementation science theories and tools, such as the Behaviour Change Wheel, is important, as research indicates that interventions developed using theories are more likely to be successful.^44^ Furthermore, in social research, theoretical frameworks such as Intersectionality or Empowerment enhance critique, clarity, and analytical depth, strengthening the rigour of studies by enabling deeper exploration of human experiences, behaviours, outcomes, and structural inequalities.^45,46^ However, the extent to which theories have been used in research into online sexual, reproductive, and transgender healthcare for LGBTQI+ youth is unexplored.

Therefore, the objective of this current study was to identify and describe recent literature on online sexual, reproductive, and transgender healthcare for LGBTQI+ youth, synthesise study findings, and make recommendations for future research. To achieve this objective, three review questions (RQs) were addressed:

(RQ1) Within literature on online sexual, reproductive, and transgender healthcare, what areas of health, health topics, types of healthcare, and online platforms have received attention for LGBTQI+ youth and where are there gaps?

(RQ2) Who are the target populations of online sexual, reproductive, and transgender healthcare research for LGBTQI+ youth and where are there gaps?

(RQ3) How, if at all, have theories, models, and frameworks been used in research into online sexual reproductive, and transgender healthcare for LGBTQI+ youth?

## Methods

### Author reflexivity

The first author (JMcL) is a white, sexual minority, cisgender woman with a background in health psychology. This study is part of her PhD into improving the sexual health of young sexual minority women. The remaining four authors have a supervisory role. The second author (CSE) is a white cisgender woman with a medical background. The joint second author (PF) is a white, sexual minority, cisgender man with a background in health psychology and is a Professor of Health Change. The third author (JG) is a white, sexual minority, cisgender woman with a clinical academic background. The last author (JMacD) is a white, cisgender woman with a background in health psychology and is a Research Fellow and JMcL's PhD Director of Studies. All authors are from the UK.

### Design

A systematic scoping review was conducted in accordance with the Joanna-Briggs Institute (JBI) methodology for scoping reviews.^43^

### Protocol registration

The protocol was published in medRxiv.^47^ See Supplementary File 2 for protocol deviations.

### Eligibility criteria

Using the Participant, Concept, Context (PCC) framework,^43^ we included research regarding LGBTQI+ youth (aged 10–35 years or using the terms youth, young people, young adults, and adolescents/teens) (Participants); online sexual, reproductive, and transgender healthcare (Concept); and studies from high-income and developed economy countries^48^ (Context) published between 2018 and 2024. See Supplementary File 3 for detailed inclusion and exclusion criteria and their rationale. Where multiple papers were published from one study, these were only included if the papers reported on unique sub-studies with distinct aims, methods, and outcomes.

High-income and developed-economy countries^48^ were selected, as access to online services can differ considerably between countries, depending on infrastructure and social welfare/protections.^49,50^ For example, as of 2019, 87% of people from developed countries had access to the internet compared to 19% of people from least-developed countries.^51^ Therefore, we focused on countries with similar contexts to the UK to ensure the findings were maximally applicable to the UK. While there is a move away from the terms “developed” and “developing”,^52^ these were still used by the United Nations as of 2023.^48^ Studies from the following countries were included: Australia; Austria; Belgium; Canada; Croatia; Cyprus; Czech Republic; Denmark; Estonia; Finland; France; Germany; Greece; Hungary; Iceland; Ireland; Italy; Japan; Latvia; Lithuania; Luxembourg; Malta; Netherlands; New Zealand; Norway; Poland; Portugal; Slovakia; Slovenia; Spain; Sweden; Switzerland; UK; and US.

Studies were limited to 2018 onwards as 2018 saw the introduction of new digital healthcare strategies in the UK^31,32^ and 2020 marked the rapid expansion of online healthcare to accommodate service provision during international COVID-19 lockdown.^33^ Additionally, by 2018, 90% of people in the UK had access to the internet^53^ and 87% of people in developed countries had access to the internet,^51^ enabling widespread use of online healthcare.

### Types of sources

Only published literature was included. Qualitative, quantitative, and mixed methods studies that were classified as original research with primary data collection were included. Studies using theory-based implementation and behavioural science for intervention development and evaluation were also considered for inclusion. Pilot and feasibility studies were included. Reviews, conference abstracts, posters, registered reports, blogs, guidelines, text and opinion papers, letters, editorials, commentaries, protocols, preprints, and doctoral and master's theses were excluded. Studies published in any language other than English were excluded, due to lack of resources to support translation.

### Search strategy

The PCC framework was used to structure the search, using only Participants and Concept. A preliminary search of MEDLINE, the Cochrane Database of Systematic Reviews, JBI Evidence Synthesis, and BMJ Open was conducted (31.01.2023) to identify articles on the topic. An analysis of the text words contained in the title and abstract of five retrieved papers^6,39,42,54,55^ was conducted to develop a full search strategy of four search strings (Sexual orientation/Gender minority; Age; Online; and Type of health care) (see Supplementary File 4). A full search was then undertaken across nine databases (22.05.2023): APA PsycInfo (ProQuest); APA PsycArticles (ProQuest); CINAHL Complete (EBSCO); MEDLINE (EBSCO); ERIC (EBSCO); British Education Index (EBSCO); Education Database (ProQuest); Computer Science Database (ProQuest); and Web of Science. An updated search was also conducted (19.08.2024).

### Study selection

All identified citations were exported to Excel files and uploaded to Rayyan^56^ and duplicates were removed. The titles and abstracts of deduplicated studies were screened by JMcL (100%) and RO (see Acknowledgements) (3%, *n* = 178) for assessment against the eligibility criteria. To prioritise the most relevant studies, the titles and abstracts screened by RO were ordered from most to least relevant using the “Compute Ratings” function within Rayyan, which uses artificial intelligence to calculate the probability of inclusion based on decision patterns.^56^ The full text of studies categorised as “Included” and “Maybe” were then assessed in detail against the eligibility criteria by JMcL (100%) and RO (10%, *n* = 20). Of the 178 articles screened by both reviewers, there was an 85% consistency. The conflicts were resolved through discussion and referral to the protocol. For the updated search, all titles and abstracts of deduplicated studies and full texts were screened by JMcL for assessment against the eligibility criteria.

### Data extraction

Data from included papers were extracted to Microsoft Excel by JMcL using a data extraction tool adapted (by JMcL) from the JBI Manual for Evidence charting table for data extraction synthesis.^43^ The tool was centred around the PCC framework for extracting relevant data for study details, RQ1, RQ2 and RQ3. The tool was reviewed by PF and JMacD, then piloted and refined using a relevant paper identified from the preliminary search.^57^ No authors were contacted to request missing or additional data. Supplementary File 5 details the data extraction process for each of the relevant variables presented in the results.

### Data analysis

Following extraction, analysis involved calculating frequency counts and percentages for study details and relevant data regarding each of the review questions. Analyses were then charted in tables and summarised narratively to provide an overview of the included papers for each of the review questions. Supplementary File 6 provides an overview of data extraction for each of the relevant variables presented in the results.

### Quality appraisal

In line with JBI scoping review methodology,^43^ a quality appraisal was not conducted. This review mapped the literature and did not analyse nor draw conclusions from the outcomes of studies.

## Results

### Study selection

**Figure 1.**
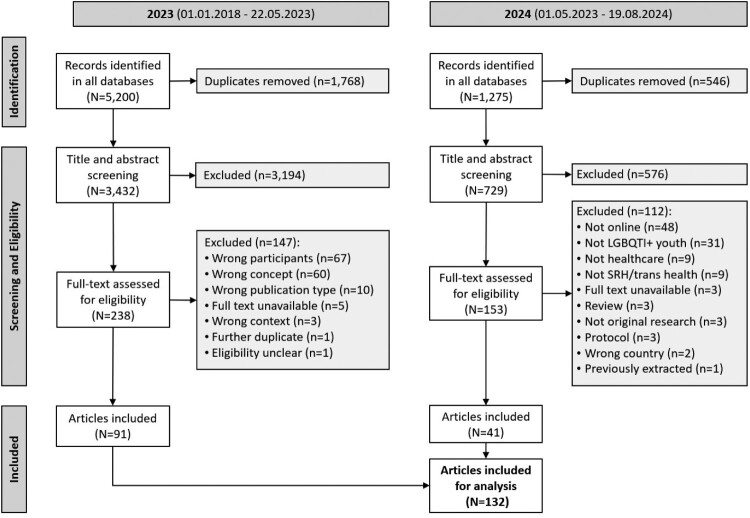
PRISMA-ScR flow chart of study inclusion and exclusion process

For the first search, there were an initial 5,200 hits and 1,768 were duplicates. The titles and abstracts of 3,432 papers were screened and categorised as “Excluded” (*n* = 3,194), “Maybe” (*n* = 138) and “Potentially Included” (*n* = 100). Full-text screening of the “Maybe” and “Potentially Included” papers identified 91 papers for inclusion. For the second search, there were an initial 1,275 results, of which 546 were duplicates. The titles and abstracts of 729 papers were screened and 576 were excluded. The full text of 153 papers was screened and 41 were included. In total, there were 132 included papers (see Supplementary File 7 for references and https://osf.io/4gfp6 for the full dataset). Figure 1 shows this process.

### Study characteristics

Table 1 summarises the study characteristics of the 132 included papers with citations. There was a gradual increase in papers published from 2018 (13/132) and 2019 (12/132) to 2023 (19/132) and 2024 (23/132) (as of August 2024). The vast majority of studies were conducted in the US (117/132), with few published in other countries including Canada (5/132), Australia (3/132), the UK (3/132), the Republic of Ireland (1/132), and Italy (1/132). Two studies were conducted in the US and another country: Canada (1/132) and Puerto Rico (1/132).

**Table 1.** Study characteristics of included papers in the scoping review

	Study characteristics	***n***	%
Publication date	2024	23	17.4
	2023	19	14.4
	2022	25	18.9
	2021	24	18.2
	2020	16	12.1
	2019	12	9.1
	2018	13	9.8
Country study was conducted in	United States of America (USA)	117	88.6
	Canada	5	3.8
	Australia	3	2.3
	United Kingdom	2	1.5
	England	1	0.8
	Italy	1	0.8
	Republic of Ireland	1	0.8
	USA and Canada	1	0.8
	USA including Puerto Rico	1	0.8
Study design	Quantitative	57	43.2
	Qualitative	41	31.1
	Qualitative and Quantitative	34	25.8
Data collection^a^	Survey(s) with closed questions	72	54.5
	Interviews	34	25.8
	Focus groups	16	12.1
	Routinely collected data analytics (e.g. time spent on apps)	14	10.6
	Survey with closed and open questions	12	9.1
	Biological samples	6	4.5
	Think aloud sessions	6	4.5
	Survey with open questions	4	3.0
	Electronic Health Record content	3	2.3
	Retroactive chart review	3	2.3
	Forum conversations	2	1.5
	Workshops	2	1.5
	‘Qualitative feedback/review’ (no detail given)	1	0.8
	Experiment (e.g. discrete choice experiment)	1	0.8
	Facebook data (e.g. no. of friends)	1	0.8
	Website content	1	0.8
	TikTok content	1	0.8
	YouTube video content	1	0.8
Healthcare type	Intervention (i.e. developed for study, e.g. custom web app)	84	63.6
	Service (i.e. already existing, e.g. dating app)	48	36.3
Real/Existing or Hypothetical	Real/Existing	123	93.2
	Hypothetical	9	6.8
Participants^b^	LGBTQI+ youth	120	90.9
	Parents/Caregivers/Guardians of LGBTQI+ youth	13	9.8
	Healthcare provider working with LGBTQI+ youth	6	4.5
	No participants	4	3.0
	Mentors of LGBTQI+ youth	2	1.5
	CBO staff working with LGBTQI+ youth	1	0.8
	Siblings of LGBTQI+ youth	1	0.8

^a^ Most studies used multiple data collection methods – all counted here separately.

^b^ Numbers exceed 100% as some studies recruited LGBTQI+ youth and either parents, healthcare providers, community-based organisation staff, or mentors of LGBTQI+ youth.

Regarding research methods, 57/132 were quantitative – most used surveys with closed questions (52/57) for data collection; other methods included routinely collected analytics (6/57), biological samples (5/57), and review of medical records (4/57); 41/132 papers were qualitative, most of which employed interviews (24/41) and/or focus groups (9/41); other methods included review of online content (5/41) and think aloud sessions (3/41); and 34/132 papers reported using both qualitative and quantitative methods, largely pairing surveys (33/34) with interviews (11/34) or focus groups (7/34), and/or examining routinely collected analytics, such as number of clicks or length of time spent on an intervention app (8/34).

Papers either reported on interventions developed for the study (e.g. custom mobile app, novel web app) (84/132) or explored existing services (e.g. dating apps, such as Grindr, or government websites) (48/132). Further, most of the papers reported on services or interventions that were real, not hypothetical, meaning the participants could interact with the online platform (122/132) (e.g. “the internet” including websites and social media, or a mobile app for HIV prevention information and support). A minority were hypothetical in that they sought perspectives on services or interventions that did not yet exist (9/132) (e.g. a potential mobile app for mentorship regarding HIV prevention). Overall, there were 79/132 real/existing interventions, 43/132 real/existing services, 5/132 hypothetical services, and 4/132 hypothetical interventions.

Concerning participants, LGBTQI+ youth were participants in most of the papers (120/132); a minority of papers recruited parents or caregivers (13/132), healthcare providers (6/132), mentors (2/132), community-based organisation staff (1/132), and siblings (1/132) of LGBTQI+ youth. Four papers did not have participants but collected data from existing services for LGBTQI+ youth (e.g. website or social media content).

Finally, 43/132 studies (all sexual health) were from one of fifteen studies with more than one paper, including MYPEEPS (7/43); HealthMpowerment (5/43); OutsmartHPV (4/43); MyChoices (3/43); PrEPTECH (3/43); SMART (3/43); GetConnected! (2/43); Girl2Girl (2/43); Guy2Guy (2/43); KeepItUp! (2/43); mSMART (2/43); MyDex (2/43); Parents ASSIST (2/43); Tough Talks (2/43); and weCare (2/43). Each of these papers reported unique studies with distinct aims and methods. The remaining 89 papers were from distinct studies.

### (RQ1) Within literature on online sexual, reproductive, and trans healthcare for LGBTQI+ youth, what areas of health, health topics, types of healthcare, and online platforms have received attention and where are there gaps?

Table 2 provides an overview of which areas of health and subsequent health topics and healthcare types have received attention regarding online healthcare for LGBTQI+ youth.

**Table 2.** Types of online sexual, reproductive, and transgender healthcare explored for LGBQTI+ youth in published literature

**Health topic**		***N***	**Healthcare type**					
			Education/ Information e.g.,	***n***	Non-clinical Support e.g.,	***n***	Clinical care e.g.,	***n***
**Sexual health (95^a^/132)**								
STI and BBV prevention (68/132)	HIV prevention	37	HIV risk reduction; HIV testing; PrEP; HIV disclosure; Safe sex; HIV treatment and prevention options; Sexual, injection, and other risk and practice correct steps for condom and injection use; PEP and PrEP as well as HIV transmission, HIV care, medication adherence; PrEP adherence; Risks of assuming a partner's HIV status; Risks of assumed monogamy in relationships; Healthy romantic relationships; Having pleasurable sexual experiences and acceptance of one's sexual orientation and gender identity; HIV transmission; How to acquire and use condoms; Prevention methods; Home HIV test results; PrEP stigma	26	Per communication; Reminders to take PrEP; Skill building (e.g., for safer sex choices, effective communication, coping skills, problem-solving); Feedback on PrEP adherence; PrEP adherence tracking; Q&A with trained professional; PrEP appointment reminder; Mentorship/Coaching; Q&A with automated responses; Couples counselling; Video call for help using HIV testing kit; Interpreting at home HIV test; PrEP appointment tracker; Goal setting for getting tested; Linkage to services	23	Home delivery of HIV self-test kit; eConsultation with healthcare provider for PrEP; Home delivery of PrEP	6
	STIs and BBV prevention	24	STIs; STI risk reduction; STI testing; HIV and STI prevention; Sexual health; HIV and STI epidemiology; Safer sex; Minority stress; Emotion regulation; Interpersonal and substance-related risk factors; Dating; Personal growth; Health and wellness; HIV stigma; Condom use and breakage; Sex pressures; PrEP	20	GPS maps for finding STI and HIV test sites and/ or PrEP services; Reminders for HIV testing; Reminder to take PrEP medication; Appointment reminder; Q&A with trained professional; Peer communication; Counselling; Skill building condom use	13	Home delivery of STI and HIV self-sample kits; eConsultation; Home delivery of condoms; Home delivery of PrEP	7
	Human papillomavirus (HPV) prevention	7	HPV risk and prevalence; HPV vaccination and effectiveness; HPV vaccination cost and insurance	7	Reminders to get vaccinated for HPV; GPS maps for finding HPV vaccination sites; Booking appointment; Links to resources (LGBTQI+ friendly providers); Q&A with healthcare provider; Skills-building strategies for talking with a provider about the vaccine	5	N/A	0
Sexual health *per se*		14^a^	Sexual health Relationships; Sex; How to access inclusive HIV testing; How to have conversations about sex; Safe sex; sexual practices and information related to their own or someone else's pleasure.	14	Building skills for having conversations about sex with parents and healthcare provider; Peer communication/ support/ advice regarding sexual health and safe sex	5	N/A	0
HIV management		10^a^	AIDS and HIV; Adherence, retention, and self-management; How often to see healthcare provider; ART (names, common side effects, doses per day)	8	ART medication reminder; Appointment reminder Feedback on ART adherence; Attention training; Q&A with trained professionals or automated response; Viral load tracker; Peer communication; Support groups	9	N/A	0
HIV stigma reduction		2	HIV stigma reduction	1	Peer discussions for stigma reduction and community building; Q&A with healthcare provider	2	N/A	0
Sexual safety		2	Safe social networking applications use; Healthy relationships; Dating, hookups, and online dating safety education	2	Skill building (online dating safety)	1	N/A	0
**Sexual health totals**		95		78		58		13
**Reproductive health (3/132)**								
Pregnancy prevention		2	Sex education; Birth control	2	Links to resources; Q&A with trained professional; Peer communication/ support	2	N/A	0
Reproductive care for cancer survivors		1	Providing reproductive care for adolescent and young adult LGBTQ cancer survivors	1	N/A	0	N/A	0
**Reproductive health totals**		3		3		2		2
**Sexual and reproductive health (5/132)**								
Sexual health and reproductive health *per se*		4	Sexual health; Sex education; Birth control; HPV prevention; Parent child communication	4	N/A	0	N/A	0
Sexual violence/ abuse		1		0	Collaborative and peer development of ideas for reducing abuse	1	N/A	0
**Sexual and reproductive health totals**		4		4		1		0
**Transgender health (30^a^/132)**								
Gender identity/ transition *per se*		14	Trans people's experiences; Gender affirming language; Gender identity	12	Peer support and communication; Coping skills for stigma; Links to resources	8	N/A	0
Gender affirming care		14	Gender affirming care; gender affirmation; medical records	3	N/A	0	eConsultation with healthcare provider for gender affirming care	12
Fertility		2	Fertility and parenthood; Human reproduction; Gender affirming medical treatment; Fertility Preservation; Benefits and risks of fertility preservation.	2	N/A	0	N/A	0
**Transgender health totals**		30		17		8		12
**Global totals**		**132**	**102**		**69**		**25**	

^a^ Numbers within ‘Area of health’ equal 133 instead of 132 as one paper^58^ was categorised as sexual health and transgender health, separately. Further, numbers wi thin ‘Health topic’ equal 134 instead of 132, as the same paper was subsequently categorised as both HIV management and sexual health *per se,* separately within sexual health, and gender identity/transition *per se* within transgender health.

^b^ The numbers for information/education, support, and clinical care do not total 132, as they were counted separately where papers explored more than one (e.g. if one paper explored information/education and support, this would be counted twice, once in the information/education column, and again in the support column.

#### Areas of health and health topics

The majority of the papers focused on sexual health (95/132, 72%), primarily STI and HIV prevention (68/95, 72%). A further 5/132 (4%) papers explored sexual and reproductive health together, exploring topics such as STI and pregnancy prevention and sexual violence and abuse. Only 3/132 (2%) papers explored reproductive health, two of which focussed on pregnancy prevention and one on improving reproductive healthcare for LGBTQI+ youth cancer survivors. The remaining 30/132 (23%) focused on transgender health, either investigating access to or receiving gender affirming care (14/30), issues of gender identity and expression (14/30), or fertility (2/30). One paper was categorised as exploring and sexual health and transgender health, as it examined the use of the internet for both sexual health and transgender health information as well as online technologies for HIV management separately.

##### Healthcare types

Most of the papers explored the provision of or engagement with education/information (102/132) and non-clinical support (69/132). Types of support included peer communication (e.g. forum discussions or social media interactions); skill building (e.g. how to use condoms or have conversations about sex); medication reminders (e.g. reminders to take PrEP); Question and answer (Q&A) with a trained professional or with automated responses; service locators (e.g. for STI/HIV testing or PrEP); personalised recommendations (e.g. tailored HIV testing frequency and PrEP use or strategies for ART adherence); counselling (e.g. couples counselling for HIV testing, and risk reduction); and mentorship for HIV support.

Only 25/132 explored clinical care, most frequently telehealth/ telemedicine (e-consultations with healthcare provider) (17/25) for gender-affirming care (12/17) and STI/HIV prevention (5/17), home delivery of STI/HIV self-sample/self-test kits (10/25), home delivery of condoms (3/25), and home delivery of PrEP (2/25). In one paper, it was not possible to discern the healthcare type, as it referred to being part of an online/eHealth HIV prevention with no further information.

##### Online platforms

**Figure 2.**
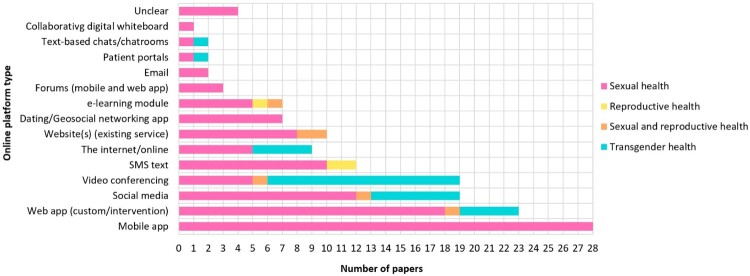
Online platforms used in sexual, reproductive, and transgender healthcare for LGBQTI+ youth. Numbers equal 152 instead of 132, as multiple papers reported on more than one online platform type

Across the 132 papers, there were various types of online platforms used (see Figure 2). The most frequently reported online platforms were mobile apps (28/132), web apps (23/132), social media (19/132), video conferencing (19/132), SMS text (12/132), “the internet” or “online” (9/132), and existing websites (10/132). Here, “online” and “the internet” were typically referred to with no further explanation of what this included.

The least commonly reported online platforms were collaborative digital whiteboards (1/132), patient portals (2/132), text-based chats (2/132), email (2/132), forums (3/132), e-learning modules (7/132), and dating/geosocial networking apps (7/132). For e-learning modules, it was unclear by which platform these were delivered. In 4/132 papers, it was unclear what online platform was used, for example, they referred to their intervention as “web-based”, “mHealth”, or “digital technologies” with no further description.

### (RQ2) Who are the target populations of online sexual, reproductive, and transgender healthcare for LGBTQI+ youth and where are there gaps?

#### Target LGBTQI+ populations

Table 3 shows the LGBTQI+ youth populations targeted in online sexual, reproductive, and transgender healthcare research by areas of health and health topics.

**Table 3.** Target LGBQTI+ youth populations for online sexual and reproductive healthcare and transgender healthcare

Health topic (*n*)		Target lesbian, gay, bisexual, queer/questioning, intersex, and other sexual and gender minorities (LGBTQI+) population (*n*)											
		LGBTQI+/ Sexual and gender minority	Lesbian, Gay, Bisexual/ Sexual minority	GBMSM^b^ (sex/ gender not specified)	GBMSM^b^ (cisgender)	GBMSM^b^ (gender inclusive assigned male at birth)	Cisgender GBMSM^b^ and trans women who have sex with men	Trans and gender diverse	Trans men/ masculine	Trans women	Sexual minority women (cis)	Sexual minority assigned female at birth (gender inclusive e.g. non-binary)	Sexual minority women (sex inclusive)
**Sexual health (95/132)^a^**													
STI and BBV prevention (68)	HIV prevention (37)	1	-	9	10	9	6	1	-	1	-	-	-
	STIs and BBV prevention (24)	-	-	6	10	5	1	-	2	-	-	-	-
	HPV^c^ prevention (7)	-	-	4	3	-	-	-	-	-	-	-	-
Sexual health per se (14)^a^		4	2	1	1	1	-	3	-	1^a^	-	-	1
HIV management (10)^a^		-	-	5	-	1	3	-	-	1^a^	-	-	-
HIV stigma reduction (2)		-	-	-	-	2	-	-	-	-	-	-	-
Sexual safety (2)		-	-	-	-	2	-	-	-	-	-	-	-
**Sexual health totals**		**5/95**	2/95	**25/95**	**24/95**	**20/95**	**10/95**	4/95	2/95	2/95	0/95	0/95	1/95
**Reproductive health (3/132)**													
Pregnancy prevention (2)		-	-	-	-	-	-	-	-	-	2	-	-
Reproductive care for cancer survivors (1)		1	-	-	-	-	-	-	-	-	-	-	-
**Reproductive health totals**		**1/3**	0/3	0/3	0/3	0/3	0/3	0/3	0/3	0/3	**2/3**	0/3	0/3
**Sexual and reproductive health (5/132)**													
Sexual health and reproductive *per se* (4)		2	-	-	-	-	-	1	-	-	-	1	-
Sexual violence/abuse (1)		1	-	-	-	-	-	-	-	-	-	-	-
**Sexual and reproductive health totals**		**3/5**	0/5	0/5	0/5	0/5	0/5	**1/5**	0/5	0/5	0/5	**1/5**	0/5
**Transgender health (30/132)**^a^													
Gender identity/ transition *per se* (14)^a^		4	-	-	-	-	-	9	-	1^a^	-	-	-
Gender affirming care (14)		-	-	-	-	-	-	14	-	-	-	-	-
Fertility (2)		-	-	-	-	-	-	2	-	-	-	-	-
**Transgender health totals**		**4/30**	0/30	0/30	0/30	0/30	0/30	25/30	0/30	1/30	0/30	0/30	0/30
**Global totals**		**13/132**	**2/132**	**25/132**	**24/132**	**20/132**	**10/132**	**30/132**	**2/132**	**2^a^**	**2**	**1**	**1**

^a^ Numbers for the column ‘Trans women’ equal 4 instead of 2, as one paper (Reback & Rünger, 2020) (targeting trans women) was classified as both sexual health and trans health separately, and, within sexual health, HIV management and sexual health *per se,* separately. Thus, one paper targeting trans women is represented three times, however, this is not reflected in the total number of trans women, where the paper is only counted once.

^b^ GBMSM = Gay, bisexual, and other men who have sex with men.

^c^ HPV = Human papillomavirus.

The most frequently targeted LGBTQI+ population was gay, bisexual and other men who have sex with men (GBMSM) (79/132), of which 34/79 papers targeted cisgender men and 20/79 targeted people “assigned male at birth” inclusive of those who identified as a different gender (e.g. non-binary); 25/79 did not specify a sex assigned at birth or gender identity. All papers that targeted GBMSM were for sexual health (largely for STI/HIV prevention, 63/79).

Further, 44/132 papers targeted trans and gender-diverse (TGD) youth, of which most were for transgender health (26/44). Of these 26 papers, 25/26 targeted TGD youth, only 1/26 targeted young trans women regarding seeking information online, and none targeted young trans men. Of the remaining papers that targeted TGD youth, 18/44 were for sexual health, and 1/44 was for sexual and reproductive health. Of the 18 sexual health papers, 12/18 targeted young trans women (10 of which also targeted young GBMSM), largely for STI and blood borne viruses (BBV) prevention (8/12) and HIV management (4/12), 4/18 targeted TGD youth, and only 2/18 targeted young trans men. No reproductive health papers targeted TGD youth.

Very few papers targeted sexual minority women (4/132), of which 2/4 targeted cisgender women for reproductive health (pregnancy prevention), and 2/4 were for sexual health (exploring information seeking), one of which targeted gender-inclusive “assigned female at birth” and one targeted people who identify as a woman. No STI and BBV prevention papers targeted sexual minority women.

Broadly, 15/132 papers targeted lesbian, gay, and bisexual (LGB)/sexual minority youth (2/15, both for sexual health) or LGBT+/sexual and gender minority youth (13/15, 5/13 for sexual health, 3/13 for sexual and reproductive 1/13 for reproductive health, and 4/13 for transgender health)

#### Target ages

Across the 132 papers, there were 50 age ranges, the most common of which were 13–18 (12/132, 9%), 15–24 (11/132, 8%), 18–29 (11/132, 8%), 18–25 (8/132, 6%), and 18–30 (8/132, 6%) years. Ten papers (8%) did not report a target age range nor the age range of the participants. The lowest minimum age was 7 and the highest minimum age was 20 and the most frequently used minimum age was 18. The lowest maximum age was 15 and the highest maximum age was 39, and the most frequently used maximum age was 24. The Appendix shows that target age ranges for transgender health clustered around younger age groups, beginning from age 7 ranging to age 26. Whereas target age ranges for sexual health clustered around older age groups, beginning from age 11 ranging to age 39. Of the 2/3 of reproductive healthcare papers that reported an age range, both targeted LGBTQI+ youth aged 14–17.

#### Intersectional factors considered

Table 4 shows PROGRESS-Plus criteria considered in recruitment of target LGBTQI+ youth for online sexual, reproductive, and transgender healthcare by areas of health and health topics. In line with the remit of the current scoping review (i.e. “LGBTQI+ youth”) “Age” was considered in all of the papers. Additionally, “Sexual orientation” was considered in most papers (101/132) but was considered notably less often in transgender health papers (4/30) than sexual health (90/95), reproductive health (3/3), and sexual and reproductive health (4/5) papers. Further, “Gender/Sex” was considered in almost all papers (130/132). However, this reflected targeting of a specific gender/sex, for example, people assigned male at birth in studies about young GBMSM in sexual health papers. Only 18/95 sexual health papers (0/3 reproductive health and 0/5 sexual and reproductive health) papers specifically targeted transgender populations. “Gender/Sex” was considered in all transgender health papers (30/30), targeting TGD youth.

**Table 4.** Intersectional factors considered in target LGBTQI+ youth populations for online sexual and reproductive healthcare and transgender healthcare

Health topic (*n*)		PROGRESS-Plus variables considered when targeting LGBTQI+ youth for online sexual and reproductive healthcare and transgender healthcare											
		PROGRESS								Plus			
		Place of residence	Race/ Ethnicity	Occupation	Gender/ Sex	Religion	Education	Socio-economic status	Social network	Features of relationships	Sexual orientation	Disability	Living with HIV
**Sexual health (95/132)^a^**													
STI and BBV prevention (68)	HIV prevention (37)	2	12	-	37	-	-	-	1	3	36	-	1
	STIs and BBV prevention (24)	3	5	-	24	-	-	-	-	1	24	-	-
	HPV^b^ prevention (7)	-	-	-	7	-	-	-	-	-	7	-	-
Sexual health per se (14)^a^		1	1	-	12^a^	-	-	-	-	-	10	-	1^a^
HIV management (10)^a^		1	4	-	10 ^a^	-	-	-	-	-	9	1	10^a^
HIV stigma reduction (2)		-	2	-	2	-	-	-	-	-	2	-	-
Sexual safety (2)		-	-	-	2	-	-	-	-	-	2	-	-
**Sexual health totals**		6/95	24/95	0/95	93/95	0/95	0/95	0/95	1/95	4/95	90/95	1/95	11/95
**Reproductive health (3/132)**													
Pregnancy prevention (2)		-	1	-	2	-	2	-	-	-	2	-	-
Reproductive care for cancer survivors (1)		-	-	-	1	-	-	-	-	-	1	-	-
**Reproductive health totals**		0/3	1/3	0/3	3/3	0/3	2/3	0/3	0/3	0/3	3/3	0/3	0/3
**Sexual and reproductive health (5/132)**													
Sexual and reproductive health *per se* (4)		-	1	-	4	-	-	-	-	-	3	-	-
Sexual violence/abuse (1)		-	-	-	1	-	-	-	-	-	1	-	-
**Sexual and reproductive health totals**		0/5	1/5	0/5	5/5	0/5	0/5	0/5	0/5	0/5	4/5	0/5	0/5
**Transgender health (30/132)**^a^													
Gender identity/ transition *per se* (14)^a^		-	1	-	14^a^	-	-	-	-	-	4	-	1^a^
Gender affirming care (14)		2	-	-	14	-	-	-	-	-	-	-	-
Fertility (2)		-	-	-	2	-	-	-	-	-	-	-	-
**Transgender health totals**		2/30	1/30	0/30	30/30	0/30	0/30	0/30	0/30	0/30	4/30	0/30	1/30
**Global totals**		**9/132**	**27/132**	0**/132**	**130**^a^**/132**	0**/132**	**2/132**	0**/132**	**1/132**	**4/132**	**101/132**	**1/132**	**11/132**

^a^ One paper (Reback & Rünger, 2020) was categorised as both sexual health and transgender health separately. Within sexual health, the paper was also subsequently categorised as HIV management and sexual health *per se,* separately. Thus, one paper is represented three times within the Gender/Sex column and the Age column. This is reflected in the totals for each area of health but it is not reflected in the global totals for these columns, where the paper is only counted once.

^b^ HPV=Human papillomavirus.

“Race/Ethnicity” was considered in 27/132 (20%) of papers, including purposeful recruitment of people of Black/African-American (22/27), Latinx/Hispanic (6/27), White/Caucasian (5/27), People of Colour (3/27), Multiracial (2/27), American Indian/Alaskan Native/Native Hawaiian/Pacific Islander (1/27), “Other” (2/27), a “range of races/ethnicities” (2/27), Asian (1/27), and Indigenous (1/27) ethnicities. However, the vast majority of papers that considered “Race/Ethnicity” were sexual health (24/27), comprising 25% of sexual health papers (24/95), all of which were studies targeting GBMSM and two also targeting young trans women. Only 1/27 was for transgender health.

“Place of Residence” was considered in 9/132 (7%) papers, including living in an urban/metropolitan area (5/9) or a rural location (4/9), and attending a clinic that serves impoverished communities (1/9). “Features of Relationships” was considered in 4/132 (3%) papers, including being or having a mentor (2/4), not being in a long-term monogamous relationship (1/4), being in a long-term non-monogamous relationship (1/4). “Education” was considered in 2/132 (2%) papers, including in high school or the equivalent (i.e. those who did not finish or dropped out) (2/2). “Disability” was considered in 1/132 paper, with a targeted focus on people with depression. “Social Networks” was considered in 1/132 paper, which focused on Facebook group membership. No papers considered Religion, Occupation or Socio-economic Status (i.e. income) in recruitment. People living with HIV were targeted in 10/132 (8%) of papers.

### (RQ3) How, if at all, have theories, models, and frameworks been used in research into online sexual, reproductive, and transgender healthcare for LGBTQI+ youth?

Table 5 provides an overview of all the frameworks used by areas of health and scientific discipline. Just over half (70/132, 53%) of the papers reported the use of at least one theory, framework or model (henceforth, theory). Most papers reported using only one theory (44/70) and 23/70 papers reported using more than one theory (two theories, 16/23; three theories, 5/23; four theories, 1/23; five theories, 1/23). Additionally, 23/70 papers reported their study to be “theory-driven” or “informed by” or having used a framework to “guide the project” or “for an in-depth exploration” with no clear replicable explanation of how this was done and 3/70 papers reported having used a framework without specifying which one.

**Table 5.** The theories, models, and frameworks used in online sexual, reproductive, and transgender healthcare for LGBTQI+ youth

**Disciplinary area**	**Theory/Framework/Model**	**Use of theory**		**Total**
		Descriptive	Applied	
**Sexual health (52/95)**				
**Cognitive and Learning Sciences** (4 theories, 4 uses, 3 studies)**** *Examines mental processes such as memory, attention, and problem-solving, and how these processes influence learning. It often integrates insights from psychology, neuroscience, and education to design effective teaching and learning tools*	**Activity theory** *(Examines human activity as a system of interactions between people and their environment, used in learning and development research)*	1	-	1
	**Cognitive Theory of Multimedia Learning** *(Explains how people learn more effectively when multimedia elements are integrated based on cognitive load principles)*	-	1	1
	**Dual Coding Theory** *(Suggests that combining verbal and visual information enhances learning and memory)*	-	1	1
	**Dual Process Theory** *(Describes two modes of thinking: fast, automatic processes (System 1) and slow, deliberate reasoning (System 2))*	-	1	1
**Communication and Media Studies** (2 theories, 3 uses, 3 studies)**** *Focuses on how individuals and groups create, transmit, and interpret messages across various media platforms. This field explores the effects of communication strategies on behaviour, attitudes, and societal norms*	**Entertainment Education** *(The strategy of embedding educational messages into entertaining media to promote behaviour change)*	-	2	2
	**Narrative Communication/Storytelling** *(Explores how stories and narratives influence understanding, attitudes, and behaviours, commonly used in health communication and education)*	-	1	1
**Healthcare and Public Health** (3 theories, 3 uses, 2 studies)**** *Focuses on improving population health and well-being through research, interventions, and systems that address healthcare delivery, chronic disease management, and evidence-based practice*	**Chronic Care Model** *(A framework for improving chronic illness care through community, self-management, and organisational support)*	-	1	1
	**Intervention Mapping** *(A step-by-step process for developing, implementing, and evaluating health interventions)*	-	1	1
	**Patient-Centred Medical Home Model** *(A healthcare delivery model focused on comprehensive, coordinated, and patient-centred care)*	-	1	1
**Implementation Science** (1 theory, 1 use, 1 study)**** *Focuses on the methods and strategies to integrate research findings and evidence-based practices into real-world settings to ensure that interventions achieve their intended outcomes in practice*	**The RE-AIM Model** *(Evaluates health interventions based on their Reach, Effectiveness, Adoption, Implementation, and Maintenance)*	-	1	1
**Information Science and Technology** (7 theories, 7 uses, 5 studies)**** *Studies the creation, organisation, management, and use of information. It also explores human interaction with information systems, technology adoption, and the design of tools to enhance usability and efficiency*	**Erdelez Model of Information Encountering** *(Focuses on the unplanned and accidental discovery of information during other activities)*	-	1	1
	**Information System Success Model** *(Evaluates the success of information systems based on factors like user satisfaction and system quality)*	-	1	1
	**Kari Conceptualisation of Information Use** *(Examines how individuals use and make sense of information in different contexts)*	-	1	1
	**The Health Information Technology Usability Evaluation Model** *(Assesses the usability of health IT systems to enhance user experience and efficiency)*	-	1	1
	**The Theoretical Framework of Information Encounters** *(Studies how people come across information serendipitously in various contexts)*	-	1	1
	**The Unified Theory of Acceptance and Use of Technology Model** *(Explains technology adoption based on factors like performance expectancy and social influence)*	-	1	1
	**The Wilson Model of Information Behaviour** *(A framework for understanding how people seek and use information based on needs and barriers)*	-	1	1
**Psychology and Behavioural Sciences** (17 theories, 49 uses, 37 studies)**** *Explores human behaviour, thoughts, and emotions, focusing on understanding and predicting individual and group actions. It includes theories on motivation, cognition, and behaviour change*	**Contingency Management** *(A behavioural strategy that uses incentives to reinforce positive behaviours)*	-	1	1
	**Information-Motivation-Behaviour Skills Model** *(Explains and predicts health behaviours by focusing on three core components: Information, Motivation, and Behavioural skills)*	-	14	14
	**Integrated Behavioural Model** *(Explains behaviour as influenced by intention, knowledge, skills, and environmental constraints)*	-	1	1
	**Minority Stress Model** *(Examines how social stressors related to minority status impact mental and physical health)*	-	2	2
	**Resilience Framework**** *(Explains how individuals or systems bounce back from adversity)*	-	1	1
	**Social-Cognitive Theory** *(Focuses on the interaction between personal, environmental, and behavioural factors in shaping behaviour)*	-	12	12
	**Social Identity Theory** *(Explores how individuals derive self-concept and behaviour from group memberships)*	-	1	1
	**Social Norms Theory** *(Focuses on the gap between perceived and actual norms to encourage healthier behaviour)*	-	1	1
	**Social-Personal (Theoretical) Framework** *(A framework adapted from Social Learning Theory adding psychosocial (e.g. affect dysregulation) and contextual risk factors (e.g. family, peer, and partner relationships) related to youth risk-taking)*	-	2	2
	**Stigma Theory** *(Explores how individuals experience stigma on a personal level, including how they internalise stigmatising attitudes and how stigma affects mental health and self-esteem)*	-	1	1
	**The Fogg Behavioural Model of Persuasive Technology** *(Explains behaviour as a function of motivation, ability, and triggers)*	-	1	1
	**The Implementation Intention Theory**** *(Suggests that forming specific “if-then” plans increases goal achievement)*	-	1	1
	**The Protection Motivation Theory** *(Explains how individuals are motivated to protect themselves based on perceived threats and coping efficacy)*	-	4	4
	**The Socio-Ecological Model** *(Examines multiple levels of influence on behaviour, from individual to societal factors)*	-	1	1
	**The Theory of Normative Social Behaviour**** *(Explores how perceived norms and expectations influence behaviour)*	-	1	1
	**The Theory of Planned Behaviour** *(Explains how attitudes, subjective norms, and perceived control influence intentions and behaviour)*	-	4	4
	**The Transtheoretical Model (Stages of Change)** *(Describes stages of behaviour change, including precontemplation, contemplation, preparation, action, and maintenance)*	-	1	1
**Sociology and Social Theory** (5 theories, 6 uses, 6 studies)**** *Studies the structure and function of societies, examining how individuals and groups interact within social, cultural, and institutional contexts, addressing systemic issues such as inequality and identity*	**Empowerment (Education) Theory** *(Focuses on fostering critical consciousness and empowering marginalised groups through education)*	-	2	2
	**Gidden's Structuration Theory** *(Explains how social structures are both the medium and outcome of social practices)*	1	1	1
	**Intersectionality** *(Analyses overlapping social identities and systemic oppression)*	-	1	1
	**Queer Theory** *(Challenges traditional notions of gender and sexuality, focusing on fluidity and diversity)*	1	-	1
	**The Pharmacopornographisation Framework** *(Analyses the intersections of biopolitics, pharmaceuticals, and pornography in shaping bodies and identities)*	1	-	1
**N/A** (0 theories 3 uses, 3 studies)	Not reported (reported using a theory but not what the theory was)	-	3	3
**Total:** 7		**Theories:** 4 **Uses:** 4 **Studies:** 4	**Theories:** 36 **Uses:** 72**Studies:** 49	**Theories:** 39 **Uses:** 75 **Studies:** 53
**Reproductive health (3/3)**				
**Psychology and Behavioural Sciences** (1 theory, 2 uses, 2 studies)	**Information-Motivation-Behaviour Skills Model** *(Explains and predicts health behaviours by focusing on three core components: Information, Motivation, and Behavioural skills)*	-	2	2
**Sociology and Social Theory** (2 theories, 2 uses, 1 study)	**Critical Race Theory** *(Examines systemic racism and its impact on marginalised groups)*	1	-	1
	**Intersectionality** *(Analyses overlapping social identities and systemic oppression)*	1	-	1
**Total:** 2	** **	**Theories:** 2 **Uses:** 2 **Studies:** 1	**Theories:** 2 **Uses:** 2 **Studies:** 2	**Theories:** 3**Uses:** 4 **Studies:** 3
**Sexual and reproductive health (3/5)**				
**Communication and Media Studies** (1 theory, 1 use, 1 study)	**The Structural Influence Model of Health Communication** *(Investigates how broader social and structural factors influence health communication outcomes)*	1	-	1
**Design Studies and Innovation Management** (2 theories, 2 uses, 2 studies)*****Focuses on creating solutions to complex problems by prioritising user needs and experiences. It often incorporates equity, inclusivity, and sustainability into the design process*	**Design Justice Framework** *(Advocates for inclusive and equitable design processes that empower marginalised communities)*	-	1	1
	**The LUMA System of Innovation** *(A toolkit for innovative problem-solving using human-centred design methods)*	-	1	1
**Sociology and Social Theory** (1 theory, 1 use, 1 study)	**Intersectionality** *(Analyses overlapping social identities and systemic oppression)*	-	1	1
**Total:** 3	** **	**Theories:** 1 **Uses:** 1 **Studies:** 1	**Theories:** 3 **Uses:** 3 **Studies:** 2	**Theories:** 4**Uses:** 4 **Studies:** 3
**Transgender health (11/30)**				
**Cognitive and Learning Sciences** (2 theories, 2 uses, 1 study)	**Constructive Alignment Model** *(Focuses on aligning teaching methods, assessments, and intended learning outcomes to maximise learning)*	-	1	1
	**Gagne's ‘9 External Events of Instruction’** *(A systematic instructional design model that outlines key steps for effective learning experiences)*	-	1	1
**Communication and Media Studies** (1 theory, 1 use, 1 study)	**Uses and Gratifications Theory** *(Examines how individuals actively seek media to satisfy specific needs or desires)*	1	-	1
**Design Studies and Innovation Management** (1 theory, 1 use, 1 study)	**Human-Centred Design** *(Focuses on designing solutions that prioritise user needs and experiences)*	-	1	1
**Implementation Science** (2 theories, 2 uses, 2 studies)	**The Iowa Model of Evidence-Based Practice** *(Guides healthcare professionals in integrating research evidence into clinical practice)*	-	1	1
	**The RE-AIM Model** *(Evaluates health interventions based on their Reach, Effectiveness, Adoption, Implementation, and Maintenance)*	-	1	1
**Psychological and Behavioural Science** (6 theories, 9 uses, 7 studies)	**Interpersonal Theory of Suicide** *(Explains suicidal behaviour based on the desire for death and the capability to enact it)*	1	1	1
	**Minority Stress Model** *(Examines how social stressors related to minority status impact mental and physical health)*	4	1	4
	**Social Identity Theory** *(Explores how individuals derive self-concept and behaviour from group memberships)*	1	-	1
	**The Socio-Ecological Model** *(Examines multiple levels of influence on behaviour, from individual to societal factors)*	1	-	1
	**The Online Disinhibition Effect**** *(Explains how people behave with less restraint online, influenced by anonymity and lack of face-to-face interaction)*	1	-	1
	**Transactional Model of Stress and Coping** *(Analyses how individuals appraise and respond to stress)*	1	-	1
**Sociology and Social Theory** (2 theories, 2 uses, 2 studies)	**Intersectionality** *(Analyses overlapping social identities and systemic oppression)*	1	1	1
	**Transgender Studies** *(Explores issues related to transgender identity, culture, and politics)*	-	1	1
**Total:** 6	**Transgender Health Theories:** 14	**Theories:** 8 **Uses:** 11 **Studies:** 6	**Theories:** 9 **Uses:** 9 **Studies:** 6	**Theories:** 14**Uses:** 17 **Studies**: 11
**Total disciplines:** 8	** **	**Theories:** 10 **Uses:** 14 **Studies:** 12	**Theories:** 47 **Uses:** 90**Studies:** 59	**Theories:** 52**Uses:** 4 **Studies:** 70

Numbers do not add up to 100% as many studies used more than one framework.

The frameworks used were highly varied: 52 frameworks were identified across the 70 papers, spanning eight disciplines: psychology and behavioural sciences (20/52 theories used across 46/70 studies); information science and technology (7/52 theories, 5/70 studies); sociology and social theory (7/52 theories, 10/70 studies); cognitive and learning sciences (6/52 theories, 4/70 studies); communication and media (4/52, 5/70 studies); design studies and innovation management (3/52 theories, 3/70 studies); healthcare and public health (3/52 theories, 2/70 studies); and implementation science (2/52 theories, 2/70 studies).

Of the 52 identified theories, the vast majority was used only once (41/52, 79%). Very few theories were used twice (7/52), four times (3/52, Protection Motivation Theory, The Theory of Planned Behaviour, Intersectionality), or six times (1/52, the Minority Stress Model). The two most commonly reported theories were both of psychology and behavioural science discipline: the Information-Motivation-Behaviour Skills Model (IMB Model), used 16 times and Social Cognitive Theory (SCT), used 12 times.

A small number of papers reported using theory in a way that was descriptive (12/70), to provide a contextual lens for understanding how social structures influence people's experiences. The theories most commonly used descriptively were the Minority Stress Model (4/12) and Intersectionality (3/12). Most of the papers reported using theory in an applied manner (59/70), for example, for development of study materials or intervention content, or to guide analyses or evaluation. Of these, most reported on an intervention (50/59) and very few on a service (9/59). This reveals that, of the 84 papers that reported on interventions, 60% applied a theory (50/84). The theories most commonly used in an applied manner were the IMB Model (16/59) and SCT (12/59).

## Discussion

### Principal results

This paper is the first to provide a comprehensive map of recent literature on online sexual, reproductive, and transgender healthcare for LGBTQI+ youth and identifies critical gaps. We identified a high volume of studies, with 132 included papers. The key findings were that the vast majority of the research was from the US. Additionally, most of the research was on sexual health, particularly HIV and STI prevention; much of this centred around the provision of or engagement with information/education and non-clinical support (e.g. reminders to get tested for HIV) and targeted young GBMSM aged 16 or 18 years and over (up to 39 years). Comparatively, there was little research into clinical care for sexual health (e.g. home delivery of STI/HIV testing kits, condoms, or PrEP and eConsultations with healthcare providers). Critically, there was very little research into reproductive health, with only two papers focusing on pregnancy prevention, and one on the delivery of inclusive reproductive care for cancer survivors. For transgender healthcare, half of the research focused on information/education and non-clinical support (e.g. peer communication/support) and the other half centred around telehealth/medicine (i.e. eConsultations with healthcare providers regarding gender affirming care). Most of this research targeted TGD youth between 7 and 26 years. Across all of the papers, a wide range of online platforms were explored, most of which were developed as novel interventions including mobile apps, websites/web apps, and some of which were existing services such as websites, social media, and geosocial networking/dating apps. Importantly, most research did not consider socio-economic demographics associated with inequalities in health (e.g. race/ethnicity, place of residence, occupation, and education) in the recruitment of LGBTQI+ youth. Finally, most papers reporting on interventions used at least one theory, model, or framework, indicating that the majority of online SRH care interventions developed and evaluated for LGBQTI+ youth draw upon theory in some way.

### Contextualisation of results and key gaps

#### Countries

**High-income countries:** This review identified a dearth of research into online sexual, reproductive, and transgender healthcare for LGBTQI+ youth outside the US. Of 34 countries eligible for inclusion, studies were only from seven: 90% (119/132) were from the US and only 11% (14/132) from Canada, Australia, the UK, the Republic of Ireland, and Italy. This indicates that our current understanding of online sexual, reproductive, and transgender healthcare for LGBTQI+ youth largely stems from one national context. The absence of studies from other countries is an important gap, as financial cost is an often-reported barrier to sexual, reproductive, and gender affirming care in the US.^59^ The US healthcare system is primarily private and significantly more expensive than other countries,^60^ whereas healthcare in the UK, Ireland, Italy, Canada, and Australia is typically funded by public taxes and free at the point of use.^61^ Therefore, conclusions drawn from the current literature such as patterns of uptake, acceptability of, and barriers to online sexual, reproductive, and gender affirming care for LGBTQI+ youth should take economic differences into consideration.

**Low and middle income countries:** Although this scoping review focussed on high-income and developed countries, there is also a pressing need for research in low-and-middle-income countries (LMIC). LGBTQI+ populations in LMIC face considerable political and social stigma and discrimination,^62–64^ adverse sexual and reproductive health outcomes,^65,66^ and barriers to healthcare.^66–68^ There are reviews into or including LMIC, such as sexual health services for GBMSM^69^ and sexual and reproductive health service uptake among LGBTQI+ populations.^70^ However, none focus on online healthcare. A systematic review into digital interventions for sexual health promotion among young people identified only 3/25 studies in LMIC and 7/25 on LGBTQI+ populations.^71^ Similarly, a review into globally digitally available sexual and reproductive toolkits designed for young people identified 4/16 focussed on LMIC and 10/16 included LMIC, and only 3/16 were tailored for LGBTQI+ youth.^72^ Although each study reports on LGBTQI+ populations and LMIC separately and the intersection of these is not specified, the low numbers indicate very little existing research into online sexual and reproductive healthcare for LGBTQI+ youth in LMIC.

**Future research:** Research across countries, including high-income countries outside of the US and LMIC is needed to widen our global understanding of online sexual, reproductive, and transgender healthcare for LGBTQI+.

#### Areas of health

This review found that the research into online sexual, reproductive, and transgender healthcare is dominated by sexual health, particularly STI and BBV prevention. Despite this, there were critical gaps within STI and BBV prevention.

**HIV management:** HIV management was under-researched and there was no literature regarding online options for partner notification.^73^ Partner notification is a core strategy for reducing the spread of STIs and BBVs^74^ and digital communication channels are increasingly being used to support this process in general populations.^75^ The lack of research into online partner notification among LGBTQI+ youth means that the uptake of, and barriers to the use of such options are unknown and opportunities to increase timely partner notification may be missed.

**Sexual wellbeing and violence/abuse:** While both sexual wellbeing and sexual violence/abuse can fall under sexual health,^76^ very few papers focused on these critical issues or specified the inclusion of them within sexual health. LGBTQI+ youth can access healthcare for both sexual wellbeing^40^ and sexual violence^41^ on online platforms designed for the general public. However, research has shown that LGBTQI+ youth can struggle to identify relevant information and legitimate sources of information online.^27,38^ Additionally, because their identities and experiences as sexual and gender minorities often differ from those of heterosexual and cisgender individuals, LGBTQI+ youth may require tailored care to meet their unique needs, particularly for complex issues such as sexual violence/abuse.^77^

**Reproductive healthcare:** The little research into online reproductive care largely focused on pregnancy prevention, exclusively targeting cisgender sexual minority women. Online reproductive healthcare is a growing field, with the delivery of telemedicine for family planning,^78^ medical abortion,^79^ and e-contraception^80^ being developed for the general population. The lack of research into reproductive health for LGBTQI+ youth is problematic, as LGBTQI+ youth, including but not limited to cisgender sexual minority women, are among the highest at risk for early and unplanned pregnancy.^3,81^ Additionally, given that young adults can include those aged 18–39, LGBTQI+ youth may require fertility preservation or assistance,^4,82^ yet there was no research into the online provision of this. This gap risks maintaining or increasing inequalities in reproductive health outcomes for LGBTQI+ youth.

**Future research:** Research into online SRH care for LGBTQI+ youth should focus on sexual health topics beyond HIV and STI prevention; in particular, important issues such as HIV management, sexual safety, sexual abuse and violence, and sexual wellbeing. Additionally, research is also needed into the delivery of and engagement with online healthcare for reproductive and fertility issues for LGBTQI+ youth.

#### Types of healthcare

**Sexual health:** The overwhelming majority of research into online sexual healthcare focussed on the use of platforms, largely mobile and web apps, to deliver targeted information and practical support for sexual health. However, very few studies have considered clinical care, such as online STI and BBV testing. This is problematic, as national online STI testing services are being designed and delivered across various countries, including the UK,^34^ Canada,^25^ Australia,^36^ and the US.^35^ The lack of research into clinical care means that such online services are being developed without an evidence base that includes the needs of LGBTQI+ youth, particularly young sexual minority women and TGD youth. This may limit the usability of online sexual health services for LGBTQI+ youth and contribute to health disparities.

**Transgender health:** Very few studies explored education/information regarding gender-affirming care for trans youth. The three studies that explored this provided no detail about what the education/information provided via virtual visits, patient portals, or “web-based sources” entailed. This is an important finding, as the internet, particularly social media, is a popular source of information about gender affirmation and transgender healthcare for TGD youth,^83^ however, such online information can often be inaccurate.^84^ This scoping review indicates TGD youth are lacking in research-based resources about such healthcare that have been developed or evaluated to ensure accuracy. This also suggests a lack of empirical research into how LGBTQI+ youth are engaging with information about gender-affirming care.

**Future research:** Within HIV and STI prevention, research is needed into clinical care delivered online that targets LGBTQI+ youth, such as the development or evaluation of online healthcare for STI and HIV testing and treatment, partner notification, and e-consultations. Additionally, research is needed into the evaluation of existing and development of novel formal educational resources for TGD youth about gender identity and gender-affirming care.

#### LGBTQI+ youth populations

**LGBTQI+ populations:** The vast majority of the research into online sexual healthcare for populations focussed on young GBMSM. While GBMSM have a disproportionately high burden of STIs/BBVs,^2^ TGD youth and young bisexual girls and women are also at high risk^3^ and are considerably under-researched for online innovations within sexual health prevention and management.

**Intersectionality:** Intersecting socio-economic demographics and characteristics associated with inequalities in health (e.g. Place of Residence, Race/Ethnicity, Occupation, Religion, Education, Socio-Economic Status^19,20^) were largely overlooked when developing targeted digital sexual, reproductive, and transgender healthcare interventions for LGBQTI+ youth. This lack of consideration of intersectionality in recruitment is problematic, as such demographics can impact access to online technology and the internet^50^ and sexual and reproductive health outcomes.^85,86^ Research which recruits only GBMSM, White, and socioeconomically advantaged LGBTQI+ youth risks masking important inequalities and limiting the applicability of findings to others in need of tailored interventions. Addressing intersectional inequalities may be fundamental in improving the sexual and reproductive health of LGBTQI+ youth.

**Age:** The wide range of minimum and maximum ages used in included studies (7–39 years) illustrates that “youth” has been operationalised loosely across the literature. This suggests that research into online sexual and transgender healthcare is inclusive of diverse developmental stages. However, age boundaries spanning four decades are problematic, as LGBTQI+ “youth” will have markedly different generational, developmental, social, sexual, health, and healthcare experiences within adolescence,^87,88^ and even more so across four decades. Research with those in their thirties is unlikely to be applicable to those in early adolescence or younger. Therefore, operationalisation of youth should be taken into consideration when drawing from the existing evidence base.

**Future research:** Research into online SRH care is urgently needed among young sexual minority women and TGD youth, particularly young trans men, to ensure that the needs of all LGBTQI+ youth can be met. Additionally, future research should consider generating target sampling frames using the PROGRESS-Plus framework for purposeful recruitment of LGBTQI+ youth from populations associated with health inequalities. Moreover, future research could adopt standardised age brackets, such as the World Health Organisation's 15–24.^89^ or the UN's 10–19,^90^ to enable transferability of study findings to populations of the same age range.

#### Theory

**Use of theory:** Over half of the studies used at least one framework, primarily for intervention development or evaluation. Additionally, theories were largely applied in a practical way to guide intervention content, data collection, or analysis. This indicates a strong evidence base, as interventions developed using theory have a higher likelihood of success.^44^ However, there was a notable lack of theories used descriptively to provide a contextual lens for research. This indicates that research into online sexual, reproductive, and transgender healthcare is focussed on designing and delivering solutions before fully conceptualising the problems they aim to address or the context in which they are situated.^44,91^ Early theoretical grounding of research can deepen understanding of the structural factors that shape health inequalities and facilitate provision of more effective interventions and services.^91,92^

**Disciplines:** Theories spanned eight disciplines; this conceptual diversity mirrors the complexity of LGBTQI+ youth's experiences and the online environments in which sexual, reproductive, and transgender healthcare are being delivered. However, there was an over-reliance on individual-level psychological theories, such as the Social Cognitive Theory, and a lack of use of theories that consider the structural and community-level influences on behaviour, such as the Socio-Ecological Model. The use of such theories could facilitate the provision of interventions and services that target multi-level factors that maintain inequalities in sexual and reproductive health outcomes for LGBTQI+ youth.^93^

Moreover, although many studies reported on novel interventions, there was very little use of theories from implementation science, limiting intervention optimisation for adoption and long-term effectiveness or understanding of how interventions might be scaled in real-world settings.^94^ Additionally, despite the focus on online healthcare, very few studies adopted technology-based theories, limiting the understanding of how digital contexts influence intervention uptake and engagement.^95^ Furthermore, no studies drew from developmental and life-course theories. Interventions with LGBTQI+ youth can impact short- to medium-term sexual health behaviours (Biello et al., 2022; Horvath et al., 2023; Reiter et al., 2023, see Supplementary File 7 for references) and LGBTQI+ adults also experience disproportionately poor sexual health outcomes.^2^ Therefore, life-course theories could aid in understanding long-term solutions for addressing inequalities in sexual health outcomes among LGBTQI+ populations.

**Future research:** Research into online sexual, reproductive, and transgender healthcare for LGBTQI+ youth should extend application of theory to initial stages for comprehensive conceptualisation of problems and later stages for contextualisation of findings. Additionally, theories that incorporate structural components, such as the Socio-Ecological Model, should be used more frequently to develop multi-layered solutions to inequalities in LGBTQI+ youth sexual and reproductive health outcomes. Equally, theories should be drawn more often from disciplines beyond psychology and behaviour change, including implementation science, information technology, and life-course and development, to facilitate problem-solving from more diverse angles.

### Implications

As services are increasingly delivered online, this scoping review makes a novel and timely contribution to the field of sexual and reproductive health – particularly amid rising anti-LGBTQI+ rhetoric and legislation in countries such as the US and UK^96,97^ – by providing the first comprehensive map of research into online sexual, reproductive, and transgender healthcare for LGBTQI+ youth. Key gaps included research outside the US; research into areas of sexual health beyond STIs and HIV, including sexual wellbeing and sexual violence/abuse; research into reproductive health; research including or focussing on young sexual minority women, non-binary people assigned female at birth, and transgender men; and research prioritising marginalised populations, such as people of minoritised ethnic backgrounds and those living in the most deprived areas. While there is a growing body of literature, the gaps identified have notable implications for a range of stakeholders. For **researchers and digital health developers**, the review underscores the need to expand research beyond HIV and STI prevention, focus on under-researched populations, and address the geographic imbalance by conducting research across countries, globally. For **sexual and reproductive healthcare providers**, this review exposes significant gaps in the evidence that informs online service provision. These gaps indicate that existing online SRH care may not be adequately addressing the needs of young sexual minority women and TGD youth and should not be assumed to offer an equitable solution to inequalities in access to care for LGBTQI+ youth. Finally, for **policymakers and funders**, the research gaps offer a clear agenda for future investigation and funding priorities. Without inclusive research, online SRH care risks unintentionally reinforcing existing health disparities among LGBTQI+ youth. By identifying critical blind spots, this review supports efforts to achieve equity in health outcomes and ensure that the needs of all LGBTQI+ youth are met.

### Strengths

First, the volume of included papers in this scoping review provided a comprehensive overview of the literature on sexual, reproductive, and transgender healthcare, depicting the breadth of research and identifying clear gaps.^98^ Further, following the JBI methodology^43^ and using nine databases^99^ for the search ensured that this scoping review was conducted in a rigorous and systematic manner and facilitated a thorough identification and mapping of the literature on online sexual, reproductive, and transgender healthcare for LGBTQI+ youth, represented by the volume of studies. Another strength of the study was the contribution of two reviewers to screening both titles/abstracts and full texts. This reduced the chance of bias^100^ and ensured that the eligibility criteria were well understood, and methods were replicable by a researcher outside the field of sexual health.

### Limitations

The volume of papers in this study classified it as a large scoping review (100+), which introduced analytical and reporting constraints.^98^ First, the breadth and heterogeneity of included studies regarding health areas and topics, online platforms, and LGBTQI+ populations limited the feasibility of conducting cross-study analyses that require behavioural and contextual specificity, such as thematic synthesis of barriers, facilitators, or acceptability.^44^ Follow-up systematic reviews with more focussed health areas, online platforms, or LGBTQI+ youth populations could be conducted to explore these.^101^ Second, the large number of included studies constrained the level of detail that could be reported within the scope of a single review, necessitating a more descriptive and quantitative synthesis, rather than in-depth discussion of individual studies. Another limitation of this study may be that only five papers’ titles, abstracts, and key words were used to specify search terms. However, no guidance on how many papers to include for identifying search terms is provided by JBI.^43^ Additionally, due to the volume of included papers, the reference lists of included papers were not searched. Therefore, while the authors’ best efforts were made to ensure that all possibly relevant studies were included in this scoping review, some papers may have been missed.

## Conclusions

While there is a wide range of research into online sexual, reproductive, and transgender healthcare, the majority of the existing research for online sexual and reproductive healthcare focusses on the perspectives of young gay, bisexual, and other men who have sex with men pertaining to HIV and STI prevention and centres around the provision of or engagement with education or information and non-clinical support, such as reminders to get tested or to take anti-retroviral medication. There are critical gaps in the literature including research focusing on reproductive healthcare, the provision of clinical care, and the perspectives of other LGBTQI+ sub-populations such as TGD youth and young sexual minority women. Moreover, within transgender healthcare specifically, there is a gap in the literature around formal sources of information about gender identity, transition, and gender-affirming care. Further, intersectional demographics and characteristics associated with inequalities in health, such as education, occupation, income, and religion, are chronically under-considered in recruitment. The PROGRESS-Plus framework could be a useful tool for targeted recruitment of diverse populations. Given the shift to the delivery of healthcare online and that LGBTQI+ youth have disproportionately poor sexual and reproductive health outcomes and low engagement with sexual and reproductive healthcare, it is vital that these research gaps are filled to ensure the needs of LGBTQI+ youth are met.

## Supplementary Material

Supplementary File 2. Deviations from the protocol

Supplementary File 1. Abbreviations and key terms

Supplementary File 5. Data extraction

Supplementary File 4. Search terms

Supplementary File 3. Inclusion and exclusion criteria

Supplementary File 7. References of included studies

Supplementary File 6. Data analysis

## Provenance statement

This paper was not commissioned and went through external peer review.

## Appendix

### Target age ranges of LGBTQI+ youth for online sexual and reproductive healthcare and transgender healthcare

Numbers equal 133 instead of 132, as one paper was categorised as both sexual health and transgender health separately and counted twice here.
