# Supplementary material for: Mapping the missing: a scoping review identifying critically underrepresented LGBTQI+ youth within online sexual, reproductive, and transgender healthcare research
Source: Sex Reprod Health Matters. 2026 May 29;33(1):2679359. doi: 10.1080/26410397.2026.2679359 (PMC13288906; doi:10.1080/26410397.2026.2679359)
Supplement: Supplementary File 2. Deviations from the protocol [file ZRHM_A_2679359_SM1073.docx]

### Supplementary File 2. Deviations from the protocol

First, the phraseology of every RQs was altered. All RQs were updated to refer to ‘transgender healthcare’ in place of ‘gender healthcare’ for a more accurate representation of the concept. Additionally, ‘developed’ countries was removed from all RQs – while this was still included in the eligibility criteria, this terminology is outdated. Further, the initial fifth research question was removed and merged with RQ2.

Second, two of the six original review questions (RQs) were removed (see Table A1) due to the high-volume of papers and subsequent heterogeneity of areas of health, online platforms, and populations which would have limited the usefulness of collating information on acceptability and barriers and facilitators. As a result, a quality appraisal was not conducted, as there were no analyses conducted to make interpretations or draw conclusions about the findings of any papers. Subsequently, “and evaluate the methodological quality of” studies was removed from the objective.

Third, a grey literature search was not conducted due to the unexpectedly high number of included studies from the database search – a grey literature search was deemed unnecessary given the high volume of peer-reviewed, published papers.

**Table A1. Changes to review questions.**

| **Protocol** | **Current study** | **Rationale** |
| --- | --- | --- |
| RQ1) What dimensions of digital SRHC and GHC for LGBTQI+ youth in high-income, developed countries have received attention in the literature and who are the target populations? | RQ1) Within recent literature on online sexual, reproductive, and transgender healthcare, what areas of health, health topics, types of healthcare, and online platforms have received attention and where are there gaps? | ‘and who were the target populations [of online sexual, reproductive and transgender healthcare]’ was removed from this question and addressed in a separate review question (RQ2) in the results. |
| RQ2) What are the characteristics of LGBTQI+ youth in research regarding digital SRHC and GHC and accessing and using digital SRHC and GHC in high-income, developed countries? | RQ2) Who are the target populations of recent research into online sexual, reproductive, and transgender healthcare for LGBTQI+ youth and where are there gaps? | The question about who was ‘accessing and using’ digital sexual and reproductive healthcare and transgender healthcare was removed, as the data available did not support answering. Additionally, the question about the ‘characteristics of LGBTQI+ youth in research’ was too close to, and replaced by ‘the target LGBTQI+ youth populations in research’ as this better represents the author’s intentions to identify which LGBTQI+ youth populations were the target of online healthcare services and interventions. |
| RQ3) What is the acceptability of digital SRHC and GHC in high-income, developed countries for LGBTQI+ youth? | Deleted |  |
| RQ4) What are the barriers and facilitators to LGBTQI+ youth accessing and using digital SRHC and GHC in high-income, developed countries? | Deleted |  |
| RQ5) How is LGBTQI+ ‘youth’ defined in digital SRHC and GHC research from high-income, developed countries? | Deleted | Age ranges were addressed under the review question about target populations. |
| RQ6) How, if at all, have theory or frameworks been used in research into digital SRHC and GHC for LGBTQI+ youth in high-income, developed countries? | RQ3) Within recent research, how, if at all, have theories, models, and frameworks been used in research into online sexual reproductive, and transgender healthcare for LGBTQI+ youth? | This remained the same but gender healthcare was replaced with transgender healthcare and digital was replaced with online, in keeping with all other review questions. |
