# Supplementary material for: Mapping the missing: a scoping review identifying critically underrepresented LGBTQI+ youth within online sexual, reproductive, and transgender healthcare research
Source: Sex Reprod Health Matters. 2026 May 29;33(1):2679359. doi: 10.1080/26410397.2026.2679359 (PMC13288906; doi:10.1080/26410397.2026.2679359)
Supplement: Supplementary File 1. Abbreviations and key terms [file ZRHM_A_2679359_SM1072.docx]

### Supplementary File 1. Abbreviations and key terms

| **Term** | **Definition (citation)** |
| --- | --- |
| **Abbreviations** | |
| GBMSM | Gay, bisexual, and other men who have sex with men |
| HIV | Human immunodeficiency virus |
| HPV | Human papillomavirus |
| LGBTQI+ | Lesbian, gay, bisexual, transgender, queer, questioning, intersex, and other sexual orientation and gender diverse and minority populations |
| PCC | Participants, Concept, and Context |
| PrEP | Pre-exposure prophylaxis |
| SRHC | Sexual and reproductive healthcare |
| STI | Sexually transmitted infection |
| UK | United Kingdom |
| UN | United Nations |
| USA | United States of America |
| WHO | World Health Organisation |
| **Key terms** | |
| Cisgender | Those whose gender identity aligns with their sex assigned at birth (Stonewall, n.d.; YoungScot, 2022) |
| Cisnormative/ Cisnormativity | The societal assumption that people are cisgender and the use of language solely relating to cisgender people, purposefully or inadvertently neglecting recognition of trans and other gender diverse people (Roy & Singh, 2024) |
| Gender affirmation | A social/appearance and/or physical/medical transition to align one’s gender expression and body with their gender identity (Diamond, 2020; Garofalo & Garvin, 2020) |
| Gender affirming care | Clinical care to treat gender dysphoria and/or support people in living in the gender that is most authentic and comfortable to them (e.g., mental health assessments; puberty blockers; menstrual suppression; hormone replacement therapy; fertility preservation or assistance, gender reassignment surgery) (Bhatt et al., 2022; Chen et al., 2016; Coleman et al., 2012; Hidalgo et al., 2013; Lee & Rosenthal, 2023; Sterling & Garcia, 2020; van de Grift, 2023) |
| Gender dysphoria | Emotional distress due to incongruence between sex and gender identity (Kuper et al., 2019) |
| Gender expression | The way in which people manifest, convey and present their gender (Diamond, 2020) |
| Gender identity | Internal sense of gender (Diamond, 2020) |
| Heteronormative | The societal assumption that people are heterosexual and the use of language solely relating to heterosexuality, purposefully or inadvertently neglecting recognition of non-heterosexual sexual identities (Roy & Singh, 2024) |
| Intersex | Those who may have the biological attributes of both sexes or whose biological attributes do not fit with societal assumptions about what constitutes male or female (Stonewall, n.d.) |
| Medical transition | Taking hormones (e.g., testosterone or oestrogen) and/or undergoing surgery to align one’s body with their gender identity (Defreyne et al., 2023; Kuper et al., 2019; Miller et al., 2019; van de Grift, 2023) |
| Partner notification | When sex partners are informed of their exposure to an STI or HIV (Ferreira et al., 2013) |
| Pre-exposure prophylaxis (PrEP) | An antiretroviral drug to reduce the risk of acquiring HIV (Spinner et al., 2016) |
| Social transition | Altering one’s gender expression, appearance, and other social aspects to match their gender identity, such as clothes, hair, name, pronouns (Diamond, 2020; Kuper et al., 2019) |
| Telemedicine | Video conferencing for conversations or counselling with a healthcare provider (Lucas et al., 2023) |
| Trans | An umbrella term for gender diverse and minority populations (Chen et al., 2016) |
| Transgender health | Living in the gender that feels most authentic and comfortable, based on the premise that sex and gender are distinct. This can involve gender affirmation and transition to align one’s body and gender expression with their gender identity (Garofalo & Garvin, 2020) |
| Transgender healthcare | Information, support, and clinical care regrading gender identity, expression, and transition. Includes but is broader than gender affirming care (Gorton & Grubb, 2014) |

**References**

Bhatt, N., Cannella, J., & Gentile, J. P. (2022). Gender-aﬃrming Care for Transgender Patients. *Innovations in Clinical Neuroscience*, *19*, 23–32.

Chen, D., Hidalgo, M. A., Leibowitz, S., Leininger, J., Simons, L., Finlayson, C., & Garofalo, R. (2016). Multidisciplinary Care for Gender-Diverse Youth: A Narrative Review and Unique Model of Gender-Affirming Care. *Transgender Health*, *1*(1), 117–123. https://doi.org/10.1089/trgh.2016.0009

Coleman, E., Bockting, W., Botzer, M., Cohen-Kettenis, P., DeCuypere, G., Feldman, J., Fraser, L., Green, J., Knudson, G., Meyer, W. J., Monstrey, S., Adler, R. K., Brown, G. R., Devor, A. H., Ehrbar, R., Ettner, R., Eyler, E., Garofalo, R., Karasic, D. H., … Zucker, K. (2012). Standards of Care for the Health of Transsexual, Transgender, and Gender-Nonconforming People, Version 7. *International Journal of Transgenderism*, *13*(4), 165–232. https://doi.org/10.1080/15532739.2011.700873

Defreyne, J., Vander Stichele, C., Iwamoto, S. J., & T’Sjoen, G. (2023). Gender-affirming hormonal therapy for transgender and gender-diverse people—A narrative review. *Best Practice and Research: Clinical Obstetrics and Gynaecology*, *86*. https://doi.org/10.1016/j.bpobgyn.2022.102296

Diamond, L. M. (2020). Gender Fluidity and Nonbinary Gender Identities Among Children and Adolescents. *Child Development Perspectives*, *14*(2), 110–115. https://doi.org/10.1111/cdep.12366

Ferreira, A., Young, T., Mathews, C., Zunza, M., & Low, N. (2013). Strategies for partner notification for sexually transmitted infections, including HIV. *Cochrane Database of Systematic Reviews*, *2013*(10). https://doi.org/10.1002/14651858.CD002843.pub2

Garofalo, E. M., & Garvin, H. M. (2020). The confusion between biological sex and gender and potential implications of misinterpretations. In *Sex Estimation of the Human Skeleton: History, Methods, and Emerging Techniques* (pp. 35–52). Elsevier. https://doi.org/10.1016/B978-0-12-815767-1.00004-3

Gorton, N., & Grubb, H. M. (2014). General, sexual, and reproductive health. In laura Erickson-Schroth (Ed.), *Trans Bodies, Trans Selves A Resource for the Transgender Community* (1st ed.). Oxford University Press. https://www.google.co.uk/books/edition/_/EuB_AwAAQBAJ?hl=en&gbpv=1

Hidalgo, M. A., Ehrensaft, D., Tishelman, A. C., Clark, L. F., Garofalo, R., Rosenthal, S. M., Spack, N. P., & Olson, J. (2013). The gender affirmative model: What we know and what we aim to learn. *Human Development*, *56*(5), 285–290. https://doi.org/10.1159/000355235

Kuper, L. E., Lindley, L., & Lopez, X. (2019). Exploring the Gender Development Histories of Children and Adolescents Presenting for Gender Affirming Medical Care. *Clinical Practice in Pediatric Psychology*, *7*(3), 217–228. https://doi.org/10.1037/cpp0000290

Lee, J. Y., & Rosenthal, S. M. (2023). Gender-Affirming Care of Transgender and Gender-Diverse Youth: Current Concepts. *Annual Review of Medicine*, *74*, 107–116. https://doi.org/https://doi.org/10.1146/annurev-med-043021- 032007

Lucas, R., Kahn, N., Bocek, K., Tordoff, D. M., Karrington, B., Richardson, L. P., & Sequeira, G. M. (2023). Telemedicine Utilization Among Transgender and Gender-Diverse Adolescents Before and After the COVID-19 Pandemic. *Telemedicine and E-Health*, *29*(9), 1304–1311. https://doi.org/10.1089/tmj.2022.0382

Miller, T. J., Wilson, S. C., Massie, J. P., Morrison, S. D., & Satterwhite, T. (2019). Breast augmentation in male-to-female transgender patients: Technical considerations and outcomes. *JPRAS Open*, *21*, 63–74. https://doi.org/10.1016/j.jpra.2019.03.003

Roy, S., & Singh, M. (2024). The Institutionalization Of Heteronormativity And Cisnormativity In Educational Institutes: A Review. *International Journal of Interdisciplinary Approaches in Psychology*, *2*(4), 571–590. https://www.psychopediajournals.com/index.php/ijiap/article/view/257

Spinner, C. D., Boesecke, C., Zink, A., Jessen, H., Stellbrink, H.-J., Rockstroh, J. K., & Esser, S. (2016). HIV pre-exposure prophylaxis (PrEP): a review of current knowledge of oral systemic HIV PrEP in humans. *Infection*, *44*(2), 151–158. https://doi.org/10.1007/s15010-015-0850-2

Sterling, J., & Garcia, M. M. (2020). Fertility preservation options for transgender individuals. *Translational Andrology and Urology*, *9*, S215–S226. https://doi.org/10.21037/tau.2019.09.28

Stonewall. (n.d.). *Easy Read Learn more about us Contents*. Stonewall. Retrieved February 17, 2023, from https://www.stonewall.org.uk/list-lgbtq-terms

van de Grift, T. C. (2023). Masculinizing and defeminizing gender-affirming surgery. *Best Practice and Research: Clinical Obstetrics and Gynaecology*, *88*. https://doi.org/10.1016/j.bpobgyn.2023.102323

YoungScot. (2022). *Gender Identity Terms*. YoungScot. https://young.scot/get-informed/gender-identity-terms
