# Supplementary material for: Mapping the missing: a scoping review identifying critically underrepresented LGBTQI+ youth within online sexual, reproductive, and transgender healthcare research
Source: Sex Reprod Health Matters. 2026 May 29;33(1):2679359. doi: 10.1080/26410397.2026.2679359 (PMC13288906; doi:10.1080/26410397.2026.2679359)
Supplement: Supplementary File 5. Data extraction [file ZRHM_A_2679359_SM1071.docx]

### Supplementary File 5. Data extraction

#### Review question 1

For RQ1, data were extracted and analysed regarding areas of health, health topic, types of healthcare, and online platforms explored. For areas of health, papers were deductively categorised as belonging to sexual, reproductive, or transgender health. For health topic, data were inductively identified from the title, aim or methods of the paper. For types of healthcare, data were deductively categorised as Education/Information, Non-clinical Support, and Clinical Care. Education/Information refers to services and interventions that impart information only typically to achieve an increase in knowledge (e.g., information about STIs/HIV; contraception; or gender expression and transition). Non-clinical Support refers to services and interventions that provide non-clinical emotional or practical support, beyond information, typically to achieve a desired outcome (e.g., peer communication for HIV stigma reduction; reminders for increased PrEP adherence; or skill building for increased skills for partner notification). Clinical care refers to services and interventions for medical care, specific to testing, diagnosing, treating, and managing sexual and reproductive health issues or gender affirming care (e.g., STI/HIV testing; uptake or maintenance of PrEP; consultations for gender affirming hormones). Finally, for online platforms, the verbatim terms used in papers by authors were extracted.  For online platforms, data were extracted from the title, research aims, or methods using the verbatim terms used in papers by authors.

#### Review question 2

For RQ2, data were extracted and analysed regarding which LGBTQI+ populations were targeted for the intervention/service and which intersectional factors were considered in eligibility or recruitment. For target LGBTQI+ populations, data were extracted from the title or methods using the verbatim terms used in papers by authors. For analysis, different terms with the same or similar meanings were combined, for example, ‘same sex attracted boys/men’, ‘gay men’, ‘men who have sex with men’, were categorised as GBMSM. Moreover, the PROGRESS+ framework (PROGRESS and other factors associated with inequalities in health topic, including Sexual orientation, Age, and Disability) (Kavanagh & Oliver, 2008; O’Neill et al., 2014) was used to identify how intersectionality was considered. We also included Living with HIV as an additional factor.

#### Review question 3

For RQ3, frequency counts and percentages were calculated for the number of papers that reported use of one or more theory, model or framework (hereby framework) and how they were used. In extraction, how frameworks were used was captured deductively, grouped into either ‘Theoretical’ or ‘Applied’. ‘Theoretical’ describes when frameworks were used to provide a contextual lens for understanding how social structures influence people’s experiences. ‘Applied’ describes when frameworks were used in an applied manner, for example, for development of study materials or intervention content, or to guide analyses or evaluation.

**Table A2. Data extraction for variables presented in the results.**

|  | **Variables (columns in Excel)** | **Data extracted** |
| --- | --- | --- |
| Study details | Author of publication | First author’s last name, et al. |
|  | Date of publication | Year of publication |
|  | Study aim/objective | Verbatim from the abstract or introduction |
|  | Study research questions/ objectives | Verbatim from the introduction |
|  | Research method/Study design | Verbatim or deductive from the methods |
|  | Data collection method | Verbatim from the methods |
|  | Analysis methods | Verbatim from the methods |
|  | Sample size | Verbatim from the methods |
|  | Eligibility/Inclusion criteria | Verbatim from the methods |
|  | Recruitment methods | Verbatim from methods (including sampling type, where participants were recruited from, how they were recruited) |
| RQ1 (Concept) | Area of health | Identified and extracted based on the focus of the study from the title, abstract, introduction, and methods. |
|  | Health topic | Identified and extracted based on the focus of the study, from the title, abstract, introduction, and methods. |
|  | Healthcare type: Education/ Information | Identified and extracted from the methods (intervention/service description) or title, abstract/introduction |
|  | Healthcare type: Education/ Information type | Verbatim from the methods explaining the intervention/service |
|  | Health care type: Non-clinical Support | Identified and extracted from the methods (intervention/service description) or title, abstract/introduction |
|  | Health care type: Non-clinical support type | Verbatim from the methods explaining the intervention/service |
|  | Health care type: Clinical care | Identified and extracted from the methods (intervention/service description) or title, abstract/introduction |
|  | Health care type: Clinical care type | Verbatim from the methods explaining the intervention/service |
|  | Online type | Identified and extracted verbatim from the title, abstract, aim, and/or methods explaining the intervention/service |
|  | Intervention/service | Identified and extracted from the methods describing the intervention/service |
|  | Real/Hypothetical | Identified and extracted from the methods describing the intervention/service |
|  | Intervention name | Extracted verbatim from title, introduction, aim, or methods |
|  | Intervention/service details | Extracted verbatim from the methods describing the intervention/service |
| RQ2 (Participants) | Target LGBTQI+ population | Identified and extracted verbatim first from the title, then the methods (participants and recruitment section), then the introduction (aims/objectives), respectively. |
|  | Target LGBTQI+ population age range | Identified and extracted first from the methods (participants and recruitment section), then the results (participant characteristics), then the introduction, respectively. |
|  | PROGRESS-Plus: Place of residence | Identified and extracted from recruitment or eligibility/inclusion criteria in the methods. Data from the results section was not extracted. |
|  | PROGRESS-Plus: Race/ Ethnicity |  |
|  | PROGRESS-Plus: Occupation |  |
|  | PROGRESS-Plus: Gender/Sex |  |
|  | PROGRESS-Plus: Religion |  |
|  | PROGRESS-Plus: Education |  |
|  | PROGRESS-Plus: Socio-economic status (income) |  |
|  | PROGRESS-Plus: Social network |  |
|  | PROGRESS-Plus: Age |  |
|  | PROGRESS-Plus: Disability |  |
|  | PROGRESS-Plus: Sexuality |  |
|  | PROGRESS-Plus: Features of relationships |  |
|  | PROGRESS-Plus: Time dependent relationships |  |
|  | Other: Living with HIV |  |
|  | Target descriptor category | Extracted verbatim from title, aim, and/or methods (categories: youth; young [LGBTQI+]; young adults; young people; teen; adolescent – from) |
|  | Participant age range | Extracted verbatim from methods |
|  | Participant age stats | Extracted verbatim from methods (including mean and standard deviation; median and interquartile range; age range percentages) |
| RQ3 | Theory/Model/Framework | Identified and extracted verbatim from the introduction, methods, analysis, and/or discussion. If a framework was mentioned without any further explanation of its relevance to the study, it was not included. |
|  | How Theory/Model/Framework was used | Identified and extracted from the methods, then introduction, then results, then discussion, respectively. |
|  | Number of frameworks used | Calculated from description of frameworks in introduction or methods |
| Context | Country | Verbatim from methods or inductive from introduction or methods |

^a^Where a study did not report data, these were extracted as N/R (not reported).
