# Supplementary material for: Mapping the missing: a scoping review identifying critically underrepresented LGBTQI+ youth within online sexual, reproductive, and transgender healthcare research
Source: Sex Reprod Health Matters. 2026 May 29;33(1):2679359. doi: 10.1080/26410397.2026.2679359 (PMC13288906; doi:10.1080/26410397.2026.2679359)
Supplement: Supplementary File 4. Search terms [file ZRHM_A_2679359_SM1070.docx]

### Supplementary File 4. Search terms

| **Search string** | **Search terms^a^** | |
| --- | --- | --- |
|  | **2023** | **2024** |
| Sexual orientation/ gender minority | LGB* OR  Lesbian OR  Gay OR  Bisexual OR  lesbians OR  gays OR  bisexuals OR  homosexual OR  homosexuals OR  Trans OR  Transgender OR  Transexual OR  Transsexual OR  “Trans men” OR  “Trans women” OR  “Trans man” OR  “Trans woman” OR  Queer OR   Questioning OR  Intersex OR  Asexual OR  Aromantic OR  Pansexual OR  “Men who have sex with men” OR  MSM OR  “Women who have sex with women” OR  WMW OR  “Women who have sex with women and men” OR  WSWM OR  “Women who have sex with men and women” OR  WSMW OR  “Men who have sex with men and women” OR  MSMW OR  “Men who have sex with women and men” OR  MSWM OR  “Gay and bisexual men who have sex with men” OR  GBMSM OR  Non-binary OR  Nonbinary OR  “Non binary” OR  “Gender non-conforming” OR  “Sexual* minority” OR  “Sexual identity minority” OR  “Sexual orientation minority” OR  “sexual* minorities” OR  “sexual orientation minorities” OR  “sexual identity minorities” OR  “sexual-identity minorities” OR  “sexual-orientation minorities” OR  “sexual* minority” OR   “sexual-identity minority” OR  “sexual-orientation minority” OR  “gender minorities” OR  “Gender minority” OR  Same-sex OR  “same sex” OR  Non-heterosexual OR  Nonheterosexual OR  Non-cisgender OR  Noncisgender OR  “Gender diverse” OR  “Gender-diverse” | LGB* OR  Lesbian OR  Gay OR  Bisexual OR  lesbians OR  gays OR  bisexuals OR  homosexual OR  homosexuals OR  Transgender OR  Transexual OR  Transsexual OR  “Trans men” OR  “Trans women” OR  “Trans man” OR  “Trans woman” OR  Queer OR   Questioning OR  Intersex OR  Asexual OR  Aromantic OR  Pansexual OR  “Men who have sex with men” OR  “Women who have sex with women” OR  “Women who have sex with women and men” OR  “Women who have sex with men and women” OR  “Men who have sex with men and women” OR  “Men who have sex with women and men” OR  “Gay and bisexual men who have sex with men” OR  GBMSM OR  Non-binary OR  Nonbinary OR  “Non binary” OR  “Gender non-conforming” OR  “Sexual* minority” OR  “Sexual identity minority” OR  “Sexual orientation minority” OR  “sexual* minorities” OR  “sexual orientation minorities” OR  “sexual identity minorities” OR  “sexual-identity minorities” OR  “sexual-orientation minorities” OR  “sexual* minority” OR   “sexual-identity minority” OR  “sexual-orientation minority” OR  “gender minorities” OR  “Gender minority” OR  Same-sex OR  “same sex” OR  Non-heterosexual OR  Nonheterosexual OR  Non-cisgender OR  Noncisgender OR  “Gender diverse” OR  “Gender-diverse”  Removed: Trans^b^;  MSM^c^;  WMW^c^;  WSWM^c^;  WSMW^c^;  MSMW^c^;  MSWM^c^ |
| Age | Youth OR  “Young people” OR  “Young adult*” OR  “Young-adult” OR  “Young person*” OR  “Young-person” OR  Teen* OR  Adolescent* OR  “Young MSM” OR  “Young GBMSM” OR  YMSM OR  YBMSM OR  young | Youth OR  “Young people” OR  “Young adult*” OR  “Young-adult” OR  “Young person*” OR  “Young-person” OR  Teen* OR  Adolescent* OR  Young  Removed: “Young MSM” ^d^;  “Young GBMSM” ^d^;  YMSM^c^;  YBMSM^c^ |
| Online | Mobile-based OR  Mobile-application OR  Mobile-App OR  “Mobile based” OR  “Mobile application” OR  “Mobile app” OR  Website OR  Web-based OR  Web-application OR  Web-app OR  “Web based” OR  “Web application” OR  “Web app” OR  Digital OR  “Digital Health” OR  eHealth OR   mHealth OR  Smartphone OR  Telehealth OR  Telemedicine OR  Telecommunication OR  “Tele communication” OR  “Tele health” OR  “Tele medicine” OR  “Tele care” OR  “Technology Enabled Care Services” OR  “TECS” OR “Artificial Intelligence” OR  Artificial-intelligence OR   online OR  internet OR  “Mobile health” OR  “electronic health” OR  “live chat” OR  “video chat” OR  “video consultation” OR  eConsult OR  eConsultation OR  “internet intervention” OR  AI | Mobile-based OR  Mobile-application OR  Mobile-App OR  “Mobile based” OR  “Mobile application” OR  “Mobile app” OR  Website OR  Web-based OR  Web-application OR  Web-app OR  “Web based” OR  “Web application” OR  “Web app” OR  Digital OR  “Digital Health” OR  eHealth OR   mHealth OR  Smartphone OR  Telehealth OR  Telemedicine OR  Telecommunication OR  “Tele communication” OR  “Tele health” OR  “Tele medicine” OR  “Tele care” OR  “Technology Enabled Care Services” OR  “TECS” OR “Artificial Intelligence” OR  Artificial-intelligence OR   online OR  internet OR  “Mobile health” OR  “electronic health” OR  “live chat” OR  “video chat” OR  “video consultation” OR  eConsult OR  eConsultation OR  “internet intervention”  Removed: AI^b^ |
| Type of health care | “Sexual health” OR  “Sexual health care” OR  “Sexual healthcare” OR  “Sexual and reproductive health” OR  “Sexual and reproductive health care” OR  “Sexual and reproductive healthcare” OR  “Reproductive health” OR  “Reproductive health care” OR  “Reproductive healthcare” STI OR  “Sexually transmitted infection” OR  STD OR  “Sexually transmitted disease” OR  HIV OR  “Human immunodeficiency virus” OR  Chlamydia OR  Gonorrh* OR  Syphilis OR  Herpes OR  Hepatitis OR  “Bacterial vaginosis” OR  “Human papilloma virus” OR  “Human papillomavirus” OR  HPV OR  “Genital warts” OR  “condyloma acuminatum” OR  Fertility  “Pregnancy management” OR  “Pregnancy prevention” OR  “Pregnancy termination” OR  “Pregnancy assistance” OR  “Fertility management” OR  “Fertility assistance” OR  “Fertility preservation” OR  “Sexual wellbeing” OR  “Sexual well-being” OR  “Sexual pleasure” OR  “Sexual violence” OR  “Sexual abuse” OR  “Sexual harassment” OR  Gender OR  “Gender identity” OR  “Gender expression” OR  “Gender transition” OR  Transition OR   “Social transition” OR  “Medical transition” OR  “Physical transition” OR  “Cross sex” OR  “gender dysphoria” OR  “Partner notification” OR  “Partner notification and management” OR  “Contact tracing” OR  “Pre-exposure prophylax*” OR  PrEP OR  “Post-exposure prophylax*” OR  Contraception OR  Condom OR  Femidom OR  “Dental dam” OR  “In vitro fertilisation” OR  “In uterine insemination” OR   IVF OR  IUI OR  “Gender affirming” OR   “Gender-affirming” OR  “Gender reaffirming” OR  “Hormone replacement” OR  “HRT” OR  “Hormone block*” OR  “Puberty block*” OR  “feminizing hormone” OR  “masculinizing hormone” OR  “feminising hormone” OR  “masculinising hormone”  “hormone treatment” OR  “hormone therapy” OR  “testosterone therapy” OR  “STI care” OR  “HIV care” OR  “Sexual health Service” OR  “sexual healthcare service” OR  “sexual health care service” OR  “Sexual health and reproduction Service” OR  "STI Intervention" OR  "HIV intervention" OR  "sexual health intervention" OR  "STI Prevention" OR  "HIV prevention" OR  "sexual respect" OR  "sexual health education" OR  "sexual health Information" OR  "sexual health promotion" OR   "STI Testing" OR  "HIV testing" OR  "STI self-sampling" OR  "HIV self-sampling" OR  “HIV self-test” OR  “HIV-self-testing” OR  "STI treatment" OR  "HIV treatment" OR  "STI test results" OR  "HIV test result" OR  "HIV test results" OR  "HIV management" OR  "STI screening" OR  "HIV screening" OR  "sexual health Assistance" OR  "sexual health advice" OR  "sexual health Support" OR  "sexual health Counselling" OR  "sexual health Therapy" OR  "sexual and reproductive health intervention" OR  "sexual health and reproduction education" OR  "sexual health and reproduction Information" OR   "sexual health and reproduction promotion" OR   "sexual health and reproduction assistance" OR  "sexual health and reproduction advice" OR  "sexual health and reproduction Support" OR  "sexual health and reproduction Counselling" OR  "sexual health and reproduction Therapy" OR  "HIV counselling" OR   "HIV therapy" OR  "HIV support" OR  "HIV advice" OR  "Gender health" OR  "gender affirming therapy" OR  "gender affirming support" OR  "gender affirming advice" OR  "Gender affirming care advice" OR  "gender affirming care support" OR  "gender health support" OR  "gender health advice" OR  “AIDS” OR  “HIV/AIDS” OR  “HIV/STI” OR  “STI/HIV” OR  “STIs/HIV” OR  “HIV/STIs” | “Sexual health” OR  “Sexual health care” OR  “Sexual healthcare” OR  “Sexual and reproductive health” OR  “Sexual and reproductive health care” OR  “Sexual and reproductive healthcare” OR  “Reproductive health” OR  “Reproductive health care” OR  “Reproductive healthcare” STI OR  “Sexually transmitted infection” OR  STD OR  “Sexually transmitted disease” OR  HIV OR  “Human immunodeficiency virus” OR  Chlamydia OR  Gonorrh* OR  Syphilis OR  Herpes OR  Hepatitis OR  “Bacterial vaginosis” OR  “Human papilloma virus” OR  “Human papillomavirus” OR  HPV OR  “Genital warts” OR  “condyloma acuminatum” OR  Fertility OR  “Pregnancy management” OR  “Pregnancy prevention” OR  “Pregnancy termination” OR  “Pregnancy assistance” OR  “Fertility management” OR  “Fertility assistance” OR  “Fertility preservation” OR  “Sexual wellbeing” OR  “Sexual well-being” OR  “Sexual pleasure” OR  “Sexual violence” OR  “Sexual abuse” OR  “Sexual harassment” OR  Gender OR  “Gender identity” OR  “Gender expression” OR  “Gender transition” OR  Transition OR   “Social transition” OR  “Medical transition” OR  “Physical transition” OR  “Cross sex” OR  “gender dysphoria” OR  “Partner notification” OR  “Partner notification and management” OR  “Contact tracing” OR  “Pre-exposure prophylax*” OR  PrEP OR  “Post-exposure prophylax*” OR  Contraception OR  Condom OR  Femidom OR  “Dental dam” OR  “In vitro fertilisation” OR  “In uterine insemination” OR   IVF OR  IUI OR  “Gender affirming” OR   “Gender-affirming” OR  “Gender reaffirming” OR  “Hormone replacement” OR  “HRT” OR  “Hormone block*” OR  “Puberty block*” OR  “feminizing hormone” OR  “masculinizing hormone” OR  “feminising hormone” OR  “masculinising hormone” OR  “hormone treatment” OR  “hormone therapy” OR  “testosterone therapy” OR  “STI care” OR  “HIV care” OR  “Sexual health Service” OR  “sexual healthcare service” OR  “sexual health care service” OR  “Sexual health and reproduction Service” OR  "STI Intervention" OR  "HIV intervention" OR  "sexual health intervention" OR  "STI Prevention" OR  "HIV prevention" OR  "sexual respect" OR  "sexual health education" OR  "sexual health Information" OR  "sexual health promotion" OR   "STI Testing" OR  "HIV testing" OR  "STI self-sampling" OR  "HIV self-sampling" OR  “HIV self-test” OR  “HIV-self-testing” OR  "STI treatment" OR  "HIV treatment" OR  "STI test results" OR  "HIV test result" OR  "HIV test results" OR  "HIV management" OR  "STI screening" OR  "HIV screening" OR  "sexual health Assistance" OR  "sexual health advice" OR  "sexual health Support" OR  "sexual health Counselling" OR  "sexual health Therapy" OR  "sexual and reproductive health intervention" OR  "sexual health and reproduction education" OR  "sexual health and reproduction Information" OR   "sexual health and reproduction promotion" OR   "sexual health and reproduction assistance" OR  "sexual health and reproduction advice" OR  "sexual health and reproduction Support" OR  "sexual health and reproduction Counselling" OR  "sexual health and reproduction Therapy" OR  "HIV counselling" OR   "HIV therapy" OR  "HIV support" OR  "HIV advice" OR  "Gender health" OR  "gender affirming therapy" OR  "gender affirming support" OR  "gender affirming advice" OR  "Gender affirming care advice" OR  "gender affirming care support" OR  "gender health support" OR  "gender health advice" OR  “AIDS” OR  “HIV/AIDS” OR  “HIV/STI” OR  “STI/HIV” OR  “STIs/HIV” OR  “HIV/STIs” |
| Limiters | Source type: Scholarly article/Academic journals  Document type: Articles  Language: English  Date: from 2018 | Source type: Scholarly article/Academic journals  Language: English  Date: from May (01^st^) 2023 |
| Text searched | MEDLINE: Full text  CINAHL: Full text  British Education Index: Full text  ERIC: Full text  APA PsycArticles: NOFT  APA PsycInfo: NOFT  Computer Science Database: NOFT  Education Database: NOFT  Web of Science (Core Collection): All | British Education Index: Abstract only; Title only  CINAHL: Abstract only; Title only  ERIC: Abstract only; Title only  MEDLINE: Abstract only; Title only  APA PsycArticles: NOFT  APA PsycAInfo: NOFT  Computer Science Database: NOFT  Education Database: NOFT  Web of Science (Core Collection): Abstract; Title |

^a^All terms were searched title/abstract/keywords. All search strings were combined with AND.

^b^Removed because too broad a term which resulted in a high volume of irrelevant responses.

^c^Removed because unnecessary; initials are always used in conjunction with full terms (e.g., women who have sex with women) which were included in search.

^d^Removed because unnecessary repetition.
