# Supplementary material for: Mapping the missing: a scoping review identifying critically underrepresented LGBTQI+ youth within online sexual, reproductive, and transgender healthcare research
Source: Sex Reprod Health Matters. 2026 May 29;33(1):2679359. doi: 10.1080/26410397.2026.2679359 (PMC13288906; doi:10.1080/26410397.2026.2679359)
Supplement: Supplementary File 3. Inclusion and exclusion criteria [file ZRHM_A_2679359_SM1068.docx]

### Supplementary Files 3. Inclusion and exclusion criteria

#### Participants

The populations of focus in this scoping review are LGBTQI+ youth.

##### LGBTQI+

LGBTQI+ included gender and sexual orientation diverse and minority identifying participants as the target population, inclusive of all terms to describe sexual minorities and gender diverse populations (e.g., non-binary, gender non-conforming, gender fluid, and gender neutral) (Stonewall, n.d.; YoungScot, 2022). Studies with a third party (e.g., parents/ healthcare provider) as the actor for an intervention and LGBTQI+ youth as the target (see Actor, Action, Context, Target, Time; (Presseau et al., 2019) were included. LGBTQI+ (youth) were selected, as they are disproportionately at risk for poor sexual and reproductive health outcomes, low sexual health help-seeking behaviour, and face considerable, often shared, barriers to in-person sexual healthcare such as stigma, discrimination, healthcare provider lack of knowledge. Studies were excluded if they did not report on findings and conclusions for LGBTQI+ participants (i.e., reported findings and conclusions for only non-LGBTQI+) or where data and conclusions for LGBTQI+ participants could not be disaggregated from non-LGBTQI+ participant data and conclusions (i.e., reported findings and conclusions for combined LGBTQI+ and non-LGBTQI+ populations).

##### Youth

Studies that categorised target populations as ‘youth’, ‘young people’, ‘young adults’ and ‘adolescents’, ‘teens’ or included participants within an age range of 10-35 years were included. The World health Organisation and United Nations define ‘youth’ as 15-24 years, ‘young people’ as 10-24 years and ‘adolescents’ as 10-19 years ​(United Nations, n.d.; World Health Organization, n.d.-a)​. However, research into online sexual, reproductive, and transgender, healthcare employ a wider age range between 10 to 35 years (e.g., 10-20 (Decker et al., 2021)​; 13-29 ​(Wood et al., 2016)​; 16-24 (Magee et al., 2012); 16-29 ​(Flanders et al., 2017); 18-26 (McRee et al., 2015) ; 15-34 ​(Step et al., 2022). Additionally, ‘youth’ is categorised in various age ranges across countries’ legal and policy frameworks and research, ranging from 10 to 29 years ​(Perovic, 2016)​. Therefore, this broad age range was selected to capture the maximum number of studies on online sexual, reproductive, and transgender healthcare for LGBTQI+ youth research. Research focusing on non-youth populations (i.e., reported findings for child or adult populations, below 10 and above 35 years of age) or where data from participants within the age range of 10-35 years could not be disaggregated from child/adult populations was excluded.

#### Concept

The concept for this scoping review was online sexual, reproductive, and transgender healthcare. Within this were four key components: online; sexual and reproductive health; transgender health; and healthcare.

##### Online

Here, ‘online’ encompassed all sexual, reproductive, and transgender healthcare delivered via internet based digital technology, including but not limited to websites; web apps; mobile apps; short messaging service (SMS); email; and video calls e.g., ​(Gibbs et al., 2022). Studies were also included if they reported on hybrid in-person and online options or reported on both online and non-online sexual, reproductive, and transgender healthcare where it was possible to disaggregate the online from the non-online data. Studies were excluded if they reported on non-online sexual, reproductive, and transgender healthcare delivered in-person or by phone or if it was not possible to disaggregate data relating to online from non-online or if the focus of the ‘online’ aspect was recruitment of participants (e.g., online recruitment to sexual, reproductive, or transgender healthcare interventions, or recruitment to online sexual, reproductive, or transgender healthcare interventions).

##### Sexual and reproductive health

Studies were included if they related to any aspect of sexual and reproductive health, such as infection and disease (e.g., STIs and BBVs), fertility and pregnancy, sexual wellbeing, and sexual safety, violence, and abuse (Mitchell et al., 2021; World Health Organization, n.d.-b).

##### Transgender health

Studies were included if they related to any aspect of transgender health, such as issues of identity, expression, transition, and affirmation (e.g., ​(Bauermeister et al., 2022; Dehaan et al., 2013; Penfold et al., 2024) Studies focusing on care for mental health (e.g., anxiety, depression) were excluded. While anxiety and depression are key mental health issues experienced by LGBTQI+ youth, particularly trans youth, this is distinct from transgender health (i.e., gender identity, expression, affirmation and transition).

##### Healthcare

Studies were included if they focused on the delivery of or engagement with any type of healthcare. Taking an inclusive approach to healthcare, this included any intervention or service aimed at preventing, treating, or managing illness or disease or promoting wellbeing related to sexual and reproductive health and transgender health for LGBTQI+ youth. Services refer to existing online sexual, reproductive, or transgender healthcare for help-seeking, such as information on websites (e.g., (Terrance Higgins Trust, 2024); advice or support via text-based conversations with peers or trained professionals such as bi-directional email, SMS text live chat (synchronous text-based chat platform), forums, social media groups (NHS Sexual Health Hertfordshire, n.d.); or clinical care, such as online STI/HIV self-sampling, partner notification, PrEP, contraception, counselling, or e-consultations (virtual/remote platforms for video or audio consultations with healthcare provider) e.g., (Lucas et al., 2023; Sumray et al., 2022)​​. Interventions refer to online strategies that have been developed to change a specific sexual and reproductive health or transgender health related behaviour(s) or outcome(s) for LGBTQI+ youth populations including education programmes to increase knowledge (e.g., ​(Mustanski et al., 2015), support for accessing or using services such as signposting to local services (e.g., ​(West et al., 2015), or novel provision of a digital version of a service that is typically delivered in person (e.g., (Blosnich et al., 2019; Shipherd et al., 2016). This included studies that focused on the provision of or engagement with online services and interventions (e.g., ​(Saewyc et al., 2008), studies that focused on changing a specific behaviour(s) or outcome(s) e.g., ​(Mustanski et al., 2018), and studies that reported on online versions of services typically delivered in person (e.g., ​(Kincaid et al., 2023).

#### Context

For this scoping review, the context was recent (2018 onward) studies from high-income and developed economy countries.

##### Recent

‘Recent’ was defined as studies published from 2018 onward (2018-2023, extended to 2024 with an updated search). This date was selected to return a collection of the most up-to-date studies conducted in the past five years. Data older than 2018 may not be relevant in the rapidly expanding and changing field of online sexual and reproductive healthcare and gender affirming care (Norman et al., 2022; S. Robinson & Johnston, 2018; Yousaf & Currie, 2021). Additionally, LGBTQI+ rights have been subject to much discussion and change over the past five years, which may impact provision of and access to sexual and reproductive healthcare and gender affirming care ​(McDermott et al., 2021).

##### High-income countries

Studies from high-income and developed economy countries as defined by the UN ​(United Nations, 2023) were included: Australia; Austria; Belgium; Canada; Croatia; Cyprus; Czech Republic; Denmark; Estonia; Finland; France; Germany; Greece; Hungary; Iceland; Ireland; Italy; Japan; Latvia; Lithuania; Luxembourg; Malta; Netherlands; New Zealand; Norway; Poland; Portugal; Slovakia; Slovenia; Spain; Sweden; Switzerland; UK; and United States of America. High-income and developed economy countries were selected, as this scoping review is part of a wider PhD aiming to improve the sexual health of LGBTQI+ youth in the UK. As online healthcare and barriers to healthcare can differ considerably between countries, depending on infrastructure and social welfare/protections for access to health care ​(Germain et al., 2015; Gottlieb et al., 2014; L. Robinson et al., 2020)​, we focused on countries with similar contexts to the UK to ensure the findings from included studies were maximally generalisable and applicable to the UK​. While there is a move away from the terms ‘developed’ and ‘developing’ (Jimba et al., 2019)​, these terms were still used by the UN as of 2023 (United Nations, 2023).

**References**

Bauermeister, J., Choi, S. K., Bruehlman-Senecal, E., Golinkoff, J., Taboada, A., Lavra, J., Ramazzini, L., Dillon, F., & Haritatos, J. (2022). An Identity-Affirming Web Application to Help Sexual and Gender Minority Youth Cope With Minority Stress: Pilot Randomized Controlled Trial. *Journal of Medical Internet Research*, *24*(8). https://doi.org/10.2196/39094

Blosnich, J. R., Rodriguez, K. L., Hruska, K. L., Kavalieratos, D., Gordon, A. J., Matza, A., Mejia, S. M., Shipherd, J. C., & Kauth, M. R. (2019). Utilization of the veterans affairs’ transgender e-consultation program by health care providers: Mixed-methods study. *JMIR Medical Informatics*, *7*(1). https://doi.org/10.2196/11695

Decker, M. J., Atyam, T. V., Zárate, C. G., Bayer, A. M., Bautista, C., & Saphir, M. (2021). Adolescents’ perceived barriers to accessing sexual and reproductive health services in California: a cross-sectional survey. *BMC Health Services Research*, *21*(1). https://doi.org/10.1186/s12913-021-07278-3

Dehaan, S., Kuper, L. E., Magee, J. C., Bigelow, L., & Mustanski, B. S. (2013). The interplay between online and offline explorations of identity, relationships, and sex: A mixed-methods study with LGBT youth. *Journal of Sex Research*, *50*(5), 421–434. https://doi.org/10.1080/00224499.2012.661489

Flanders, C. E., Pragg, L., Dobinson, C., & Logie, C. (2017). Young sexual minority women’s use of the internet and other digital technologies for sexual health information seeking. *Canadian Journal of Human Sexuality*, *26*(1), 17–25. https://doi.org/10.3138/cjhs.261-A2

Germain, A., Sen, G., Garcia-Moreno, C., & Shankar, M. (2015). Advancing sexual and reproductive health and rights in low- and middle-income countries: Implications for the post-2015 global development agenda. *Global Public Health*, *10*(2), 137–148. https://doi.org/10.1080/17441692.2014.986177

Gibbs, J., Solomon, D., Jackson, L., Mullick, S., Burns, F., & Shahmanesh, M. (2022). Measuring and evaluating sexual health in the era of digital health: challenges and opportunities. *Sexual Health*, *19*(4), 336–345. https://doi.org/10.1071/SH22068

Gottlieb, S. L., Low, N., Newman, L. M., Bolan, G., Kamb, M., & Broutet, N. (2014). Toward global prevention of sexually transmitted infections (STIs): The need for STI vaccines. *Vaccine*, *32*(14), 1527–1535. https://doi.org/10.1016/j.vaccine.2013.07.087

Jimba, M., Fujimura, M. S., & Ong, K. I. C. (2019). Developing country: an outdated term in The Lancet. *The Lancet*, *394*, 918. https://doi.org/http://dx.doi.org/10.1016/ S0140-6736(19)32004-5

Kincaid, R., Gibbs, J., Dalrymple, J., Henderson, L., Frankis, J., & Estcourt, C. (2023). Delivering HIV prevention medication online: Findings from a qualitative study exploring the acceptability of an online HIV pre-exposure prophylaxis (PrEP) care pathway among service users and healthcare professionals. *DIGITAL HEALTH*, *9*. https://doi.org/10.1177/20552076231217816

Lucas, R., Kahn, N., Bocek, K., Tordoff, D. M., Karrington, B., Richardson, L. P., & Sequeira, G. M. (2023). Telemedicine Utilization Among Transgender and Gender-Diverse Adolescents Before and After the COVID-19 Pandemic. *Telemedicine and E-Health*, *29*(9), 1304–1311. https://doi.org/10.1089/tmj.2022.0382

Magee, J. C., Bigelow, L., DeHaan, S., & Mustanski, B. S. (2012). Sexual Health Information Seeking Online: A Mixed-Methods Study Among Lesbian, Gay, Bisexual, and Transgender Young People. *Health Education and Behavior*, *39*(3), 276–289. https://doi.org/10.1177/1090198111401384

McDermott, E., Nelson, R., & Weeks, H. (2021). The politics of LGBT+ health inequality: Conclusions from a UK scoping review. *International Journal of Environmental Research and Public Health*, *18*(2), 1–35. https://doi.org/10.3390/ijerph18020826

McRee, A., Esber, A., & Reiter, P. L. (2015). Acceptability of Home‐Based Chlamydia And Gonorrhea Testing Among a National Sample Of Sexual Minority Young Adults. *Perspectives on Sexual and Reproductive Health*, *47*(1), 3–10. https://doi.org/10.1363/47e2715

Mitchell, K. R., Lewis, R., O’Sullivan, L. F., & Fortenberry, D. J. (2021). What is sexual wellbeing and why does it matter for public health? *The Lancet Public Health*, *6*(8), e608–e613. https://doi.org/10.1016/S2468-2667(21)00099-2

Mustanski, B., Greene, G. J., Ryan, D., & Whitton, S. W. (2015). Feasibility, Acceptability, and Initial Efficacy of an Online Sexual Health Promotion Program for LGBT Youth: The Queer Sex Ed Intervention. *The Journal of Sex Research*, *52*(2), 220–230. https://doi.org/10.1080/00224499.2013.867924

Mustanski, B., Parsons, J. T., Sullivan, P. S., Madkins, K., Rosenberg, E., & Swann, G. (2018). Biomedical and Behavioral Outcomes of Keep It Up!: An eHealth HIV Prevention Program RCT. *American Journal of Preventive Medicine*, *55*(2), 151–158. https://doi.org/10.1016/j.amepre.2018.04.026

NHS Sexual Health Hertfordshire. (n.d.). *Chat sexual health* . Retrieved June 10, 2024, from https://www.sexualhealthhertfordshire.clch.nhs.uk/support/chat-sexual-health

Norman, G., Mason, T., Dumville, J. C., Bower, P., Wilson, P., & Cullum, N. (2022). Approaches to enabling rapid evaluation of innovations in health and social care: a scoping review of evidence from high-income countries. *BMJ Open*, *12*(12). https://doi.org/10.1136/bmjopen-2022-064345

Penfold, A., Callaghan, P., & Urry, K. (2024). Online Communities and Identity: Experiences of LGBTQIA+ Emerging Adults Engaging With LGBTQIA+ Online Content During the COVID-19 Pandemic. *Psychology of Popular Media*. https://doi.org/10.1037/ppm0000529

Perovic, B. (2016). DEFINING YOUTH IN CONTEMPORARY NATIONAL LEGAL AND POLICY FRAMEWORKS ACROSS EUROPE. In *European Union*. https://pjp-eu.coe.int/documents/42128013/47261653/Analytical+paper+Youth+Age+Bojana+Perovic+4.4.16.pdf/eb59c5e2-45d8-4e70-b672-f8de0a5ca08c

Presseau, J., McCleary, N., Lorencatto, F., Patey, A. M., Grimshaw, J. M., & Francis, J. J. (2019). Action, actor, context, target, time (AACTT): A framework for specifying behaviour. *Implementation Science*, *14*(1). https://doi.org/10.1186/s13012-019-0951-x

Robinson, L., Schulz, J., Blank, G., Ragnedda, M., Ono, H., Hogan, B., Mesch, G., Cotten, S. R., Kretchmer, S. B., Hale, T. M., Drabowicz, T., Yan, P., Wellman, B., Harper, M.-G., Quan-Haase, A., Dunn, H. S., Casilli, A. A., Tubaro, P., Carveth, R., … Khilnani, A. (2020). Digital inequalities and the COVID-19 pandemic 2.0: Implications of legacy digital inequalities. *First Monday*, *25*(7), 1–27. https://doi.org/https://doi.org/10.5210/fm.v25i7.10842

Robinson, S., & Johnston, P. (2018). Scotland’s Digital Health & Care Strategy: Enabling, Connecting & Empowering. In *Scottish Government*. https://www.gov.scot/binaries/content/documents/govscot/publications/strategy-plan/2018/04/scotlands-digital-health-care-strategy-enabling-connecting-empowering/documents/00534657-pdf/00534657-pdf/govscot%3Adocument/00534657.pdf

Saewyc, E. M., Poon, C. S., Homma, Y., & Skay, C. L. (2008). Stigma management? The links between enacted stigma and teen pregnancy trends among gay, lesbian, and bisexual students in British Columbia. *The Canadian Journal of Human Sexuality :*, *17*(3), 123–139.

Shipherd, J. C., Kauth, M. R., & Matza, A. (2016). Nationwide Interdisciplinary E-Consultation on Transgender Care in the Veterans Health Administration. *Telemedicine and E-Health*, *22*(12), 1008–1012. https://doi.org/10.1089/tmj.2016.0013

Step, M. M., Smith, J. M. M., Lewis, S. A., & Avery, A. K. (2022). Using the Positive Peers Mobile App to Improve Clinical Outcomes for Young People With HIV: Prospective Observational Cohort Comparison. *JMIR MHealth and UHealth*, *10*(9). https://doi.org/10.2196/37868

Stonewall. (n.d.). *Easy Read Learn more about us Contents*. Stonewall. Retrieved February 17, 2023, from https://www.stonewall.org.uk/list-lgbtq-terms

Sumray, K., Lloyd, K. C., Estcourt, C. S., Burns, F., & Gibbs, J. (2022). Access to, usage and clinical outcomes of, online postal sexually transmitted infection services: a scoping review. *Sexually Transmitted Infections*, *98*(7), 528–535. https://doi.org/10.1136/sextrans-2021-055376

Terrance Higgins Trust. (2024). *HIV and sexual health*. https://www.tht.org.uk/sexual-health

United Nations. (n.d.). *Global Issues: Youth*. United Nations. Retrieved May 20, 2025, from https://www.un.org/en/global-issues/youth

United Nations. (2023). World Economic Situation and Prospects. In *United Nations*. https://desapublications.un.org/publications/world-economic-situation-and-prospects-2023

West, R., Okecha, E., & Forbes, K. (2015). P52 Keeping “app” to date: using geolocation apps to signpost to local sexual health services. *Sexually Transmitted Infections*, *91*(Suppl 1), A32.3-A33. https://doi.org/10.1136/sextrans-2015-052126.96

Wood, S. M., Salas-Humara, C., & Dowshen, N. L. (2016). Human Immunodeficiency Virus, Other Sexually Transmitted Infections, and Sexual and Reproductive Health in Lesbian, Gay, Bisexual, Transgender Youth. *Pediatric Clinics of North America*, *63*(6), 1027–1055. https://doi.org/10.1016/j.pcl.2016.07.006

World Health Organization. (n.d.-a). *Adolescent health*. World Health Organization. Retrieved May 17, 2024, from https://www.who.int/health-topics/adolescent-health#tab=tab_1

World Health Organization. (n.d.-b). *Defining sexual health*. World Health Organization. Retrieved May 17, 2024, from https://www.who.int/teams/sexual-and-reproductive-health-and-research/key-areas-of-work/sexual-health/defining-sexual-health

YoungScot. (2022). *Gender Identity Terms*. YoungScot. https://young.scot/get-informed/gender-identity-terms

Yousaf, H., & Currie, S. (2021). Enabling, Connecting and Empowering: Care in the Digital Age. In *Scottish Government*. https://www.gov.scot/binaries/content/documents/govscot/publications/strategy-plan/2021/10/scotlands-digital-health-care-strategy/documents/enabling-connecting-empowering-care-digital-age/enabling-connecting-empowering-care-digital-age/govscot%3Adocument
