# Supplementary material for: Mapping the missing: a scoping review identifying critically underrepresented LGBTQI+ youth within online sexual, reproductive, and transgender healthcare research
Source: Sex Reprod Health Matters. 2026 May 29;33(1):2679359. doi: 10.1080/26410397.2026.2679359 (PMC13288906; doi:10.1080/26410397.2026.2679359)
Supplement: Supplementary File 7. References of included studies [file ZRHM_A_2679359_SM1067.docx]

### Appendix 7. References of included studies

| # | **Reference** |
| --- | --- |
| **Search 1: 91 included studies (2018-2023)** | |
| 1 | Albury, K., Dietzel, C., Pym, T., Vivienne, S., & Cook, T. (2024). Not your unicorn: Trans dating app users' negotiations of personal safety and sexual health. In *Social Perspectives on Trans Health* (pp. 72-86). Routledge. |
| 2 | Anderson, A., Karczmar, A., Kuhns, L. M., Garofalo, R., Radix, A., Bruce, J., ... & Schnall, R. (2022). A qualitative study to inform adaptation of MyPEEPS mobile for transmasculine youth. *Journal of Health Care for the Poor and Underserved*, *33*(1), 301-316. |
| 3 | Andrzejewski, J., Rasberry, C. N., Mustanski, B., & Steiner, R. J. (2020). Sexual and reproductive health web sites: an analysis of content for sexual and gender minority youth. *American Journal of Health Promotion*, *34*(4), 393-401. |
| 4 | Apple, D. E., Lett, E., Wood, S., Freeman Baber, K., Chuo, J., Schwartz, L. A., Petsis, D., Faust, H., & Dowshen, N. (2022). Acceptability of Telehealth for Gender-Affirming Care in Transgender and Gender Diverse Youth and Their Caregivers. *Transgender Health*, *7*(2), 159–164. <https://doi.org/10.1089/trgh.2020.0166> |
| 5 | Arayasirikul, S., Trujillo, D., Turner, C. M., Le, V., & Wilson, E. C. (2019). Implementing a digital HIV care navigation intervention (Health ENAV): Protocol for a feasibility study. *JMIR Research Protocols*, *8*(11). <https://doi.org/10.2196/16406> |
| 6 | Asiago-Reddy, E. A., McPeak, J., Scarpa, R., Braksmajer, A., Ruszkowski, N., McMahon, J., & London, A. S. (2022). Perceived access to PrEP as a critical step in engagement: A qualitative analysis and discrete choice experiment among young men who have sex with men. *PLoS ONE*, *17*(1 January). <https://doi.org/10.1371/journal.pone.0258530> |
| 7 | Austin, A., Craig, S. L., Navega, N., & McInroy, L. B. (2020). It’s my safe space: The life-saving role of the internet in the lives of transgender and gender diverse youth. *International Journal of Transgender Health*, *21*(1), 33–44. <https://doi.org/10.1080/15532739.2019.1700202> |
| 8 | Baker, D. P., Ussher, G. R., & Rimes, K. A. (2021). Development of a text-based chatroom HIV prevention and confidence-building intervention for same-sex attracted young males in South England. *Journal of HIV/AIDS and Social Services*, *20*(3), 262–270. <https://doi.org/10.1080/15381501.2021.1962473> |
| 9 | Baker, A. M., Jahn, J. L., Tan, A. S. L., Katz-Wise, S. L., Viswanath, K., Bishop, R. A., & Agénor, M. (2021). Sexual Health Information Sources, Needs, and Preferences of Young Adult Sexual Minority Cisgender Women and Non-binary Individuals Assigned Female at Birth. *Sexuality Research and Social Policy*, *18*, 775–787. <https://doi.org/10.1007/s13178-020-00501-6/Published> |
| 10 | Barry, M. C., Threats, M., Blackburn, N. A., LeGrand, S., Dong, W., Pulley, D. V., Sallabank, G., Harper, G. W., Hightow-Weidman, L. B., Bauermeister, J. A., & Muessig, K. E. (2018). “Stay strong! keep ya head up! move on! it gets better!!!!”: resilience processes in the healthMpowerment online intervention of young black gay, bisexual and other men who have sex with men. *AIDS Care - Psychological and Socio-Medical Aspects of AIDS/HIV*, *30*(sup5), S27–S38. <https://doi.org/10.1080/09540121.2018.1510106> |
| 11 | Basaran, A. M. B., Christensen, J. L., Miller, L. C., Appleby, P. R., & Read, S. J. (2019). The Relationship Between Social Norms and Sexual Risk-Reduction Intentions and Behavior Among Men Who Have Sex With Men: Findings From an eHealth Intervention. *Psychology of Addictive Behaviors*. <https://doi.org/10.1037/adb0000467> |
| 12 | Bauermeister, J. A., Muessig, K. E., LeGrand, S., Flores, D. D., Choi, S. K., Dong, W., Sallabank, G., & Hightow-Weidman, L. B. (2019a). HIV and Sexuality Stigma Reduction Through Engagement in Online Forums: Results from the HealthMPowerment Intervention. *AIDS and Behavior*, *23*(3), 742–752. <https://doi.org/10.1007/s10461-018-2256-5> |
| 13 | Bauermeister, J. A., Tingler, R. C., Demers, M., Connochie, D., Gillard, G., Shaver, J., Chavanduka, T., & Harper, G. W. (2019b). Acceptability and Preliminary Efficacy of an Online HIV Prevention Intervention for Single Young Men Who Have Sex with Men Seeking Partners Online: The myDEx Project. *AIDS and Behavior*, *23*(11), 3064–3077. <https://doi.org/10.1007/s10461-019-02426-7> |
| 14 | Bauermeister, J., Choi, S. K., Bruehlman-Senecal, E., Golinkoff, J., Taboada, A., Lavra, J., Ramazzini, L., Dillon, F., & Haritatos, J. (2022). An Identity-Affirming Web Application to Help Sexual and Gender Minority Youth Cope With Minority Stress: Pilot Randomized Controlled Trial. *Journal of Medical Internet Research*, *24*(8). <https://doi.org/10.2196/39094> |
| 15 | Berger, M. N., Taba, M., Marino, J. L., Lim, M. S. C., Cooper, S. C., Lewis, L., Albury, K., Chung, K. S. K., Bateson, D., & Skinner, S. R. (2021). Social media’s role in support networks among LGBTQ adolescents: a qualitative study. *Sexual Health*, *18*(5), 444. <https://doi.org/10.1071/SH21110_CO> |
| 16 | Bible, J., Kaplan, A., Lieberman, L., & Goldfarb, E. (2022). A retrospective analysis of sex education messages received by LGB youth. *Journal of LGBT Youth*, *19*(3), 287–306. <https://doi.org/10.1080/19361653.2020.1819509> |
| 17 | Biello, K. B., Daddario, S. R., Hill-Rorie, J., Futterman, D., Sullivan, P. S., Hightow-Weidman, L., Jones, J., Mimiaga, M. J., & Mayer, K. H. (2022). Uptake and Acceptability of MyChoices: Results of a Pilot RCT of a Mobile App Designed to Increase HIV Testing and PrEP Uptake Among Young American MSM. *AIDS and Behavior*, *26*(12), 3981–3990. <https://doi.org/10.1007/s10461-022-03724-3> |
| 18 | Biello, K. B., Horvitz, C., Mullin, S., Mayer, K. H., Scott, H., Coleman, K., Dormitzer, J., Norelli, J., Hightow-Weidman, L., Sullivan, P., Mimiaga, M. J., Buchbinder, S., Bojan, K., Futterman, D., Emmanuel, P., & Liu, A. (2021). HIV self-testing and STI self-collection via mobile apps: Experiences from two pilot randomized controlled trials of young men who have sex with men. *MHealth*, *7*. <https://doi.org/10.21037/mhealth-20-70> |
| 19 | Biello, K. B., Marrow, E., Mimiaga, M. J., Sullivan, P., Hightow-Weidman, L., & Mayer, K. H. (2019). A mobile-based app (Mychoices) to increase uptake of HIV testing and pre-exposure prophylaxis by young men who have sex with men: Protocol for a pilot randomized controlled trial. *JMIR Research Protocols*, *8*(1). <https://doi.org/10.2196/10694> |
| 20 | Blackburn, N. A., Dong, W., Threats, M., Barry, M., LeGrand, S., Hightow-Weidman, L. B., Soni, K., Pulley, D. V., Bauermeister, J. A., & Muessig, K. (2021). Building Community in the HIV Online Intervention Space: Lessons From the HealthMPowerment Intervention. *Health Education and Behavior*, *48*(5), 604–614. <https://doi.org/10.1177/10901981211003859> |
| 21 | Block, R. G., Sampson, A., Gagliardi, J., Augusto, B., Santiago-Datil, W., Schabath, M. B., Vadaparampil, S. T., & Quinn, G. P. (2022). The LOvE ECHO Training: Developing a Web-Based LGBTQ Cultural Competency Training Module for Oncology Allied Health Professionals. *Journal of Adolescent and Young Adult Oncology*, *11*(6), 556–563. <https://doi.org/10.1089/jayao.2021.0159> |
| 22 | Bonett, S., Connochie, D., Golinkoff, J. M., Horvath, K. J., & Bauermeister, J. A. (2018). Paradata analysis of an ehealth HIV testing intervention for young men who have sex with men. *AIDS Education and Prevention*, *30*(5), 434–447. <https://doi.org/10.1521/aeap.2018.30.5.434> |
| 23 | Bradford, N. J., DeWitt, J., Decker, J., Berg, D. R., Spencer, K. G., & Ross, M. W. (2019). Sex education and transgender youth: ‘Trust Means Material By and For Queer and Trans People.’ *Sex Education*, *19*(1), 84–98. <https://doi.org/10.1080/14681811.2018.1478808> |
| 24 | Brothers, J., Hosek, S., Keckler, K., Anderson, P. L., Xiong, D., Liu, H., & Huhn, G. (2022). The ATEAM study: Advances in technology to enhance PrEP adherence monitoring (ATEAM) among young men who have sex with men. *Clinical and Translational Science*, *15*(12), 2947–2957. <https://doi.org/10.1111/cts.13414> |
| 25 | Caldarera, A. M., Vitiello, B., Bechis, D., & Baietto, C. (2023). Promoting sexual health in transgender and gender diverse adolescents through an online sexuality psychoeducation program for parents: A case study. *Clinical Child Psychology and Psychiatry*, *28*(3), 1038–1052. <https://doi.org/10.1177/13591045231160641> |
| 26 | Chenneville, T., Drake, H., Gabbidon, K., Rodriguez, C., & Hightow-Weidman, L. (2021). Bijou: Engaging Young MSM in HIV Care Using a Mobile Health Strategy. *Journal of the International Association of Providers of AIDS Care*, *20*. <https://doi.org/10.1177/23259582211030805> |
| 27 | Cho, H., Powell, D., Pichon, A., Thai, J., Bruce, J., Kuhns, L. M., Garofalo, R., & Schnall, R. (2018). A mobile health intervention for HIV prevention among racially and ethnically diverse young men: Usability evaluation. *JMIR MHealth and UHealth*, *6*(9). <https://doi.org/10.2196/11450> |
| 28 | Choi, S. K., Golinkoff, J., Michna, M., Connochie, D., & Bauermeister, J. (2022). Correlates of Engagement Within an Online HIV Prevention Intervention for Single Young Men Who Have Sex With Men: Randomized Controlled Trial. *JMIR Public Health and Surveillance*, *8*(6). <https://doi.org/10.2196/33867> |
| 29 | Cordoba, E., Idnay, B., Garofalo, R., Kuhns, L. M., Pearson, C., Bruce, J., Batey, D. S., Radix, A., Belkind, U., Hidalgo, M. A., Hirshfield, S., Rodriguez, R. G., & Schnall, R. (2021). Examining the Information Systems Success (ISS) of a mobile sexual health app (MyPEEPS Mobile) from the perspective of very young men who have sex with men (YMSM). *International Journal of Medical Informatics*, *153*. <https://doi.org/10.1016/j.ijmedinf.2021.104529> |
| 30 | Dworkin, M., Chakraborty, A., Lee, S., Monahan, C., Hightow-Weidman, L., Garofalo, R., Qato, D., & Jimenez, A. (2018). A realistic talking human embodied agent mobile phone intervention to promote HIV medication adherence and retention in care in young HIV-positive African American men who have sex with men: Qualitative study. *JMIR MHealth and UHealth*, *6*(7). <https://doi.org/10.2196/10211> |
| 31 | Fields, E. L., Long, A., Dangerfield, D. T., Morgan, A., Uzzi, M., Arrington-Sanders, R., & Jennings, J. M. (2020). There’s an App for That: Using Geosocial Networking Apps to Access Young Black Gay, Bisexual, and other MSM at Risk for HIV. *American Journal of Health Promotion*, *34*(1), 42–51. <https://doi.org/10.1177/0890117119865112> |
| 32 | Fields, E. L., Thornton, N., Long, A., Morgan, A., Uzzi, M., Arrington-Sanders, R., & Jennings, J. M. (2021). Young black MSM’s exposures to and discussions about PrEP while navigating geosocial networking apps. *Journal of LGBT Youth*, *18*(1), 23–39. <https://doi.org/10.1080/19361653.2019.1700205> |
| 33 | Fish, J. N., Williams, N. D., McInroy, L. B., Paceley, M. S., Edsall, R. N., Devadas, J., Henderson, S. B., & Levine, D. S. (2022). Q Chat Space: Assessing the Feasibility and Acceptability of an Internet-Based Support Program for LGBTQ Youth. *Prevention Science*, *23*(1), 130–141. <https://doi.org/10.1007/s11121-021-01291-y> |
| 34 | Flanders, C. E., dinh, ren n., Pragg, L., Dobinson, C., & Logie, C. H. (2021). Young Sexual Minority Women’s Evaluation Processes of Online and Digital Sexual Health Information. *Health Communication*, *36*(10), 1286–1294. <https://doi.org/10.1080/10410236.2020.1751381> |
| 35 | Flores, D. D., Hennessy, K., Rosario, A., Chung, J., Wood, S., Kershaw, T., Villarruel, A., & Bauermeister, J. (2022). Parents ASSIST: Acceptability and feasibility of a video-based educational series for sexuality-inclusive communication between parents and gay, bisexual, and queer sons. *International Journal of Environmental Research and Public Health*, *19*(1). <https://doi.org/10.3390/ijerph19010379> |
| 36 | Flores, D. D., Rosario, A. A., Bond, K. T., Villarruel, A. M., & Bauermeister, J. A. (2020). Parents ASSIST (Advancing Supportive and Sexuality-Inclusive Sex Talks): Iterative Development of a Sex Communication Video Series for Parents of Gay, Bisexual, and Queer Male Adolescents. *Journal of Family Nursing*, *26*(2), 90–101. <https://doi.org/10.1177/1074840719897905> |
| 37 | Fontenot, H. B., Rosenberger, J. G., McNair, K. T., Mayer, K. H., & Zimet, G. (2019). Perspectives and preferences for a mobile health tool designed to facilitate HPV vaccination among young men who have sex with men. *Human Vaccines and Immunotherapeutics*, *15*(7–8), 1815–1823. <https://doi.org/10.1080/21645515.2019.1568156> |
| 38 | Fontenot, H. B., White, B. P., Rosenberger, J. G., Lacasse, H., Rutirasiri, C., Mayer, K. H., & Zimet, G. (2020). Mobile App Strategy to Facilitate Human Papillomavirus Vaccination among Young Men Who Have Sex with Men: Pilot Intervention Study. *Journal of Medical Internet Research*, *22*(11). <https://doi.org/10.2196/22878> |
| 39 | Frye, V., Nandi, V., Hirshfield, S., Chiasson, M. A., Wilton, L., Usher, D., Hoover, D. R., & Koblin, B. A. (2020). Randomized Controlled Trial of an Intervention to Match Young Black Men and Transwomen Who Have Sex with Men or Transwomen to HIV Testing Options in New York City (All about Me). *Journal of Acquired Immune Deficiency Syndromes*, *83*(1), 31–36. <https://doi.org/10.1097/QAI.0000000000002223> |
| 40 | Gannon, B., Davis, R., Kuhns, L. M., Rodriguez, R. G., Garofalo, R., & Schnall, R. (2020). A mobile sexual health app on empowerment, education, and prevention for young adult men (MyPEEPS Mobile): Acceptability and usability evaluation. *JMIR Formative Research*, *4*(4). <https://doi.org/10.2196/17901> |
| 41 | Gerend, M. A., Madkins, K., Crosby, S., Korpak, A. K., Phillips, G. L., Bass, M., Houlberg, M., & Mustanski, B. (2021). Evaluation of a Text Messaging-Based Human Papillomavirus Vaccination Intervention for Young Sexual Minority Men: Results from a Pilot Randomized Controlled Trial. *Annals of Behavioral Medicine*, *55*(4), 321–332. <https://doi.org/10.1093/abm/kaaa056> |
| 42 | Hedrick, H. R., Glover, N. T., Guerriero, J. T., Connelly, K. J., & Moyer, D. N. (2022). A New Virtual Reality: Benefits and Barriers to Providing Pediatric Gender-Affirming Health Care Through Telehealth. *Transgender Health*, *7*(2), 144–149. <https://doi.org/10.1089/trgh.2020.0159> |
| 43 | Hightow-Weidman, L. B., LeGrand, S., Muessig, K. E., Simmons, R. A., Soni, K., Choi, S. K., Kirschke-Schwartz, H., & Egger, J. R. (2019). A Randomized Trial of an Online Risk Reduction Intervention for Young Black MSM. *AIDS and Behavior*, *23*(5), 1166–1177. <https://doi.org/10.1007/s10461-018-2289-9> |
| 44 | Hightow-Weidman, L. B., Muessig, K., Soberano, Z., Rosso, M. T., Currie, A., Adams Larsen, M., Knudtson, K., & Vecchio, A. (2022). Tough Talks Virtual Simulation HIV Disclosure Intervention for Young Men Who Have Sex with Men: Development and Usability Testing. *JMIR Formative Research*, *6*(9). <https://doi.org/10.2196/38354> |
| 45 | Hightow-Weidman, L., Muessig, K. E., Egger, J. R., Vecchio, A., & Platt, A. (2021). Epic Allies: A Gamified Mobile App to Improve Engagement in HIV Care and Antiretroviral Adherence among Young Men Who have Sex with Men. *AIDS and Behavior*, *25*(8), 2599–2617. <https://doi.org/10.1007/s10461-021-03222-y> |
| 46 | Hightow-Weidman, L., Muessig, K., Knudtson, K., Srivatsa, M., Lawrence, E., LeGrand, S., Hotten, A., & Hosek, S. (2018). A gamified smartphone app to support engagement in care and medication adherence for HIV-positive young men who have sex with men (AllyQuest): Development and pilot study. *JMIR Public Health and Surveillance*, *4*(4). <https://doi.org/10.2196/publichealth.8923> |
| 47 | Houston, E., Fadardi, J. S., Harawa, N. T., Argueta, C., & Mukherjee, S. (2021). Individualized web-based attention training with evidence-based counseling to address HIV treatment adherence and psychological distress: exploratory cohort study. *JMIR Mental Health*, *8*(1). <https://doi.org/10.2196/18328> |
| 48 | Jackman, K. M., Latkin, C. A., Maksut, J. L., Trent, M. E., Sanchez, T. H., & Baral, S. D. (2020). Patient Portals as Highly Acceptable Tools to Support HIV Preventative Behaviors Among Adolescent and Young Sexual Minority Men. *Journal of Adolescent Health*, *67*(2), 278–281. <https://doi.org/10.1016/j.jadohealth.2020.03.029> |
| 49 | Kaufman, M. R., Casella, A., Wiginton, J. M., Xu, W., DuBois, D. L., Arrington-Sanders, R., Simon, J., & Levine, D. (2020). Mentoring young african american men and transgender women who have sex with men on sexual health: Formative research for an HIV mobile health intervention for mentors. *JMIR Formative Research*, *4*(12). <https://doi.org/10.2196/17317> |
| 50 | Kuhns, L. M., Hereth, J., Garofalo, R., Hidalgo, M., Johnson, A. K., Schnall, R., Reisner, S. L., Belzer, M., & Mimiaga, M. J. (2021). A uniquely targeted, mobile app-based HIV prevention intervention for young transgender women: Adaptation and usability study. *Journal of Medical Internet Research*, *23*(3). <https://doi.org/10.2196/21839> |
| 51 | Lee, J. J., Aguirre, J., Munguia, L., Robles, G., Ramirez Hernandez, K., Ramirez, J. I., Leyva Vera, C. A., & Duran, M. C. (2022a). Engagement of Latino immigrant men who have sex with men for HIV prevention through eHealth: preferences across social media platforms. *Ethnicity and Health*, *27*(7), 1684–1697. https://doi.org/10.1080/13557858.2021.1943322 |
| 52 | Lee, J. Y., Eimicke, T., Rehm, J. L., Connelly, K. J., & Roberts, S. A. (2022b). Providing Gender-Affirmative Care during the Severe Acute Respiratory Syndrome Coronavirus 2 Pandemic Era: Experiences and Perspectives from Pediatric Endocrinologists in the United States. *Transgender Health*, *7*(2), 170–174. https://doi.org/10.1089/trgh.2020.0151 |
| 53 | Lee-Foon, N. K., Logie, C. H., Siddiqi, A., & Grace, D. (2021). “I just trust what Google says, it’s the Bible”: Exploring young, Black gay and other men who have sex with men’s evaluation of sexual health information sources in Toronto, Canada. *Canadian Journal of Human Sexuality*, *29*(3), 275–288. <https://doi.org/10.3138/CJHS.2020-0026> |
| 54 | Li, D. H., Moskowitz, D. A., Macapagal, K., Saber, R., & Mustanski, B. (2020). Using Intervention Mapping to Developmentally Adapt an Online HIV Risk Reduction Program for Adolescent Men Who Have Sex with Men. *Prevention Science*, *21*(7), 885–897. <https://doi.org/10.1007/s11121-020-01148-w> |
| 55 | Liu, A. Y., Vittinghoff, E., Von Felten, P., Rivet Amico, K., Anderson, P. L., Lester, R., Andrew, E., Estes, I., Serrano, P., Brothers, J., Buchbinder, S., Hosek, S., & Fuchs, J. D. (2019). Randomized Controlled Trial of a Mobile Health Intervention to Promote Retention and Adherence to Preexposure Prophylaxis among Young People at Risk for Human Immunodeficiency Virus: The EPIC Study. *Clinical Infectious Diseases*, *68*(12), 2010–2017. <https://doi.org/10.1093/cid/ciy810> |
| 56 | Lucas, R., Kahn, N., Bocek, K., Tordoff, D. M., Karrington, B., Richardson, L. P., & Sequeira, G. M. (2023). Telemedicine Utilization Among Transgender and Gender-Diverse Adolescents Before and After the COVID-19 Pandemic. *Telemedicine and E-Health*, *29*(9), 1304–1311. <https://doi.org/10.1089/tmj.2022.0382> |
| 57 | Madkins, K., Moskowitz, D. A., Moran, K., Dellucci, T. V., & Mustanski, B. (2019). Measuring acceptability and engagement of the keep it up! internet-based hiv prevention randomized controlled trial for young men who have sex with men. *AIDS Education and Prevention*, *31*(4), 287–305. <https://doi.org/10.1521/aeap.2019.31.4.287> |
| 58 | Martino, W., Omercajic, K., & Cumming-Potvin, W. (2021). YouTube as a site of desubjugation for trans and nonbinary youth: pedagogical potentialities and the limits of whiteness. *Pedagogy, Culture and Society*, *29*(5), 753–772. <https://doi.org/10.1080/14681366.2021.1912156> |
| 59 | McRee, A. L., Shoben, A., Bauermeister, J. A., Katz, M. L., Paskett, E. D., & Reiter, P. L. (2018). Outsmart HPV: Acceptability and short-term effects of a web-based HPV vaccination intervention for young adult gay and bisexual men. *Vaccine*, *36*(52), 8158–8164. <https://doi.org/10.1016/j.vaccine.2018.01.009> |
| 60 | Menza, T. W., Choi, S. K., Legrand, S., Muessig, K., & Hightow-Weidman, L. (2018). Correlates of Self-Reported Viral Suppression among HIV-Positive, Young, Black Men Who Have Sex with Men Participating in a Randomized Controlled Trial of An Internet-Based HIV Prevention Intervention. *Sexually Transmitted Diseases*, *45*(2), 118–126. <https://doi.org/10.1097/OLQ.0000000000000705> |
| 61 | Mitchell, J. T., Burns, C. M., Atkinson, B., Cottrell, M., Frye, J. K., McKellar, M. S., Kashuba, A. D. M., McClernon, F. J., & Okeke, N. L. (2022). Feasibility, Acceptability, and Preliminary Efficacy of a Gamified Mobile Health Contingency Management Intervention for PrEP Adherence Among Black MSM. *AIDS and Behavior*, *26*(10), 3311–3324. <https://doi.org/10.1007/s10461-022-03675-9> |
| 62 | Mitchell, J. T., Legrand, S., Hightow-Weidman, L. B., McKellar, M. S., Kashuba, A. D. M., Cottrell, M., McLaurin, T., Satapathy, G., & McClernon, F. J. (2018). Smartphone-based contingency management intervention to improve pre-exposure prophylaxis adherence: pilot trial. *JMIR MHealth and UHealth*, *6*(9). <https://doi.org/10.2196/10456> |
| 63 | Mustanski, B., Parsons, J. T., Sullivan, P. S., Madkins, K., Rosenberg, E., & Swann, G. (2018). Biomedical and Behavioral Outcomes of Keep It Up!: An eHealth HIV Prevention Program RCT. *American Journal of Preventive Medicine*, *55*(2), 151–158. <https://doi.org/10.1016/j.amepre.2018.04.026> |
| 64 | Mustanski, B., Saber, R., Macapagal, K., Matson, M., Laber, E., Rodrgiuez-Diaz, C., Moran, K. O., Carrion, A., Moskowitz, D. A., & Newcomb, M. E. (2023). Effectiveness of the SMART Sex Ed program among 13–18 year old English and Spanish speaking adolescent men who have sex with men. *AIDS and Behavior*, *27*(2), 733–744. <https://doi.org/10.1007/s10461-022-03806-2> |
| 65 | Nelson, K. M., Perry, N. S., Stout, C. D., Dunsiger, S. I., & Carey, M. P. (2022). The Young Men and Media Study: A Pilot Randomized Controlled Trial of a Community-Informed, Online HIV Prevention Intervention for 14–17-Year-Old Sexual Minority Males. *AIDS and Behavior*, *26*(2), 569–583. <https://doi.org/10.1007/s10461-021-03412-8> |
| 66 | Newcomb, M. E., Swann, G., Macapagal, K., Sarno, E. L., Whitton, S. W., & Mustanski, B. (2023). Biomedical and Behavioral Outcomes of 2GETHER: A Randomized Controlled Trial of a Telehealth HIV Prevention Program for Young Male Couples. *Journal of Consulting and Clinical Psychology*, *91*(9), 505–520. <https://doi.org/10.1037/ccp0000823> |
| 67 | Poquiz, J. L., Shrodes, A., Garofalo, R., Chen, D., & Coyne, C. A. (2022). Supporting Pride, Activism, Resiliency, and Community: A Telemedicine-Based Group for Youth with Intersecting Gender and Racial Minority Identities. *Transgender Health*, *7*(2), 179–184. <https://doi.org/10.1089/trgh.2020.0152> |
| 68 | Reback, C. J., & Rünger, D. (2020). Technology use to facilitate health care among young adult transgender women living with HIV. *AIDS Care - Psychological and Socio-Medical Aspects of AIDS/HIV*, *32*(6), 785–792. <https://doi.org/10.1080/09540121.2019.1653439> |
| 69 | Refugio, O. N., Kimble, M. M., Silva, C. L., Lykens, J. E., Bannister, C., & Klausner, J. D. (2019). Brief Report: PrEPTECH: A Telehealth-Based Initiation Program for HIV Pre-exposure Prophylaxis in Young Men of Color Who Have Sex with Men. A Pilot Study of Feasibility. *Journal of Acquired Immune Deficiency Syndromes*, *80*(1), 40–45. <https://doi.org/10.1097/QAI.0000000000001873> |
| 70 | Reiter, P. L., Katz, M. L., Bauermeister, J. A., Shoben, A. B., Paskett, E. D., & McRee, A. L. (2018). Increasing human papillomavirus vaccination among young gay and bisexual men: A randomized pilot trial of the outsmart HPV intervention. *LGBT Health*, *5*(5), 325–329. https://doi.org/10.1089/lgbt.2018.0059 |
| 71 | Reiter, P. L., Gower, A. L., Kiss, D. E., Shoben, A. B., Katz, M. L., Bauermeister, J. A., Paskett, E. D., & McRee, A. L. (2022). Effects of a web-based HPV vaccination intervention on cognitive outcomes among young gay, bisexual, and other men who have sex with men. *Human Vaccines and Immunotherapeutics*, *18*(6). https://doi.org/10.1080/21645515.2022.2114261 |
| 72 | Rhodes, S. D., Tanner, A. E., Mann-Jackson, L., Alonzo, J., Song, E. Y., Smart, B. D., Garcia, M., McCoy, T. P., Schafer, K. R., & Wilkin, A. M. (2022). Outcomes From a Randomized Trial of a Bilingual mHealth Social Media Intervention to Increase Care Engagement Among Young Gay, Bisexual, and Other Men Who Have Sex With Men and Transgender Women With HIV. *Health Education and Behavior*, *49*(6), 975–984. <https://doi.org/10.1177/10901981221125400> |
| 73 | Russell, M. R., Rogers, R. L., Rosenthal, S. M., & Lee, J. Y. (2022). Increasing Access to Care for Transgender/Gender Diverse Youth Using Telehealth: A Quality Improvement Project. *Telemedicine and E-Health*, *28*(6), 847–857. <https://doi.org/10.1089/tmj.2021.0268> |
| 74 | Schnall, R., Kuhns, L. M., Hidalgo, M. A., Powell, D., Thai, J., Hirshfield, S., Pearson, C., Ignacio, M., Bruce, J., Batey, D. S., Radix, A., Belkind, U., & Garofalo, R. (2018). Adaptation of a group-based HIV risk reduction intervention to a mobile app for young sexual minority men. *AIDS Education and Prevention*, *30*(6), 449–462. <https://doi.org/10.1521/aeap.2018.30.6.449> |
| 75 | Schnall, R., Kuhns, L. M., Pearson, C., Batey, D. S., Bruce, J., Hidalgo, M. A., Hirshfield, S., Janulis, P., Jia, H., Radix, A., Belkind, U., Rodriguez, R. G., & Garofalo, R. (2022). Efficacy of MyPEEPS Mobile, an HIV Prevention Intervention Using Mobile Technology, on Reducing Sexual Risk among Same-Sex Attracted Adolescent Males: A Randomized Clinical Trial. *JAMA Network Open*, *5*(9), E2231853. <https://doi.org/10.1001/jamanetworkopen.2022.31853> |
| 76 | Selkie, E., Adkins, V., Masters, E., Bajpai, A., & Shumer, D. (2020). Transgender Adolescents’ Uses of Social Media for Social Support. *Journal of Adolescent Health*, *66*(3), 275–280. <https://doi.org/10.1016/j.jadohealth.2019.08.011> |
| 77 | Sequeira, G. M., Kidd, K. M., Coulter, R. W. S., Miller, E., Fortenberry, D., Garofalo, R., Richardson, L. P., & Ray, K. N. (2021). Transgender Youths’ Perspectives on Telehealth for Delivery of Gender-Affirming Care. *Journal of Adolescent Health*, *68*(6), 1207–1210. <https://doi.org/10.1016/j.jadohealth.2020.08.028> |
| 78 | Sethness, J. L., Sequeira, G. M., Kidd, K. M., Evans, Y. N., Lin, Y. H., Pratt, W., Christakis, D., Richardson, L. P., & Kahn, N. F. (2024). Guardian Reasons for Accessing Their Transgender and Gender-Diverse Adolescent’s Patient Portal Account. *Journal of Adolescent Health*, *75*(3), 516–518. <https://doi.org/10.1016/j.jadohealth.2024.06.002> |
| 79 | Sharek, D., McCann, E., & Huntley-Moore, S. (2021). The design and development of an online education program for families of trans young people. *Journal of LGBT Youth*, *18*(2), 188–210. <https://doi.org/10.1080/19361653.2020.1712296> |
| 80 | Silva, C., Fung, A., Irvine, M. A., Ziabakhsh, S., & Hursh, B. E. (2021). Usability of virtual visits for the routine clinical care of trans youth during the covid-19 pandemic: Youth and caregiver perspectives. *International Journal of Environmental Research and Public Health*, *18*(21). <https://doi.org/10.3390/ijerph182111321> |
| 81 | Stafylis, C., Vavala, G., Wang, Q., McLeman, B., Lemley, S. M., Young, S. D., Xie, H., Matthews, A. G., Oden, N., Revoredo, L., Shmueli-Blumberg, D., Hichborn, E. G., McKelle, E., Moran, L. M., Jacobs, P., Marsch, L. A., & Klausner, J. D. (2022). Relative Effectiveness of Social Media, Dating Apps, and Information Search Sites in Promoting HIV Self-testing: Observational Cohort Study. *JMIR Formative Research*, *6*(9), e35648. <https://doi.org/10.2196/35648> |
| 82 | Stephenson, R., Todd, K., Kahle, E., Sullivan, S. P., Miller-Perusse, M., Sharma, A., & Horvath, K. J. (2020). Project Moxie: Results of a Feasibility Study of a Telehealth Intervention to Increase HIV Testing Among Binary and Nonbinary Transgender Youth. *AIDS and Behavior*, *24*(5), 1517–1530. <https://doi.org/10.1007/s10461-019-02741-z> |
| 83 | Tanner, A. E., Mann-Jackson, L., Song, E. Y., Alonzo, J., Schafer, K. R., Ware, S., Horridge, D. N., Garcia, J. M., Bell, J., Hall, E. A., Baker, L. S., & Rhodes, S. D. (2020). Supporting Health Among Young Men Who Have Sex With Men and Transgender Women With HIV: Lessons Learned From Implementing the weCare Intervention. *Health Promotion Practice*, *21*(5), 755–763. <https://doi.org/10.1177/1524839920936241> |
| 84 | Threats, M., & Bond, K. (2021). HIV information acquisition and use among young black men who have sex with men who use the internet: Mixed methods study. *Journal of Medical Internet Research*, *23*(5). <https://doi.org/10.2196/22986> |
| 85 | Tolosa-Kline, A., Yom-Tov, E., Hoffman, C., Walker-Baban, C., & Lewis, F. M. T. (2021). Trojan Horse: An Analysis of Targeted Advertising to Reduce Sexually Transmitted Diseases Among YMSM. *Health Education and Behavior*, *48*(5), 637–650. <https://doi.org/10.1177/10901981211000312> |
| 87 | Ventuneac, A., Li, D. H., Mongrella, M. C., Moskowitz, D. A., Weingardt, K. R., Brown, C. H., Parsons, J. T., & Mustanski, B. (2020). Exploring Potential Implementation Barriers and Facilitators of the SMART Program: A Stepped-Care Package of eHealth HIV Prevention Interventions for Adolescent Men Who Have Sex with Men. *Sexuality Research and Social Policy*, *17*(3), 378–388. <https://doi.org/10.1007/s13178-019-00402-3> |
| 88 | Weitzman, P. F., Zhou, Y., Kogelman, L., Rodarte, S., Vicente, S. R., & Levkoff, S. E. (2021). MHealth for pre-exposure prophylaxis adherence by young adult men who have sex with men. *MHealth*, *7*. <https://doi.org/10.21037/mhealth-20-51> |
| 89 | Ybarra, M. L., Liu, W., Prescott, T. L., Phillips, G., & Mustanski, B. (2018). The Effect of a Text Messaging Based HIV Prevention Program on Sexual Minority Male Youths: A National Evaluation of Information, Motivation and Behavioral Skills in a Randomized Controlled Trial of Guy2Guy. *AIDS and Behavior*, *22*(10), 3335–3344. <https://doi.org/10.1007/s10461-018-2118-1> |
| 90 | Ybarra, M., Goodenow, C., Rosario, M., Saewyc, E., & Prescott, T. (2021). An mHealth intervention for pregnancy prevention for LGB teens: An RCT. *Pediatrics*, *147*(3). <https://doi.org/10.1542/PEDS.2020-013607> |
| 91 | Ybarra, M. L., Price-Feeney, M., Prescott, T., Goodenow, C., Saewyc, E., & Rosario, M. (2020). Girl2Girl: How to develop a salient pregnancy prevention program for cisgender sexual minority adolescent girls. *Journal of Adolescence*, *85*, 41–58. <https://doi.org/10.1016/j.adolescence.2020.09.006> |
| 92 | Young, L. E., Fujimoto, K., & Schneider, J. A. (2018). HIV Prevention and Sex Behaviors as Organizing Mechanisms in a Facebook Group Affiliation Network Among Young Black Men Who Have Sex with Men. *AIDS and Behavior*, *22*(10), 3324–3334. <https://doi.org/10.1007/s10461-018-2087-4> |
| **Search 2: 41 included studies (2023-2024)** | |
| 92 | Adedoja, D., Kuhns, L. M., Radix, A., Garofalo, R., Brin, M., & Schnall, R. (2024). MyPEEPS Mobile App for HIV Prevention Among Transmasculine Youth: Adaptation Through Community-Based Feedback and Usability Evaluation. *JMIR Formative Research*, *8*. <https://doi.org/10.2196/56561> |
| 93 | Bauermeister, J. A., Horvath, K. J., Lin, W. Y., Golinkoff, J. M., Claude, K. F., Dowshen, N., Castillo, M., Sullivan, P. S., Paul, M., Hightow-Weidman, L., & Stephenson, R. (2024). Enhancing routine HIV and STI testing among young men who have sex with men: primary outcomes of the get connected clinical randomized trial (ATN 139). *BMC Public Health*, *24*(1). <https://doi.org/10.1186/s12889-024-18522-w> |
| 94 | Boskey, E. R., Quint, M., Xu, R., Kremen, J., Estrada, C., Tham, R., Kane, K., & Reisner, S. L. (2023). Gender Affirmation–Related Information-Seeking Behaviors in a Diverse Sample of Transgender and Gender-Diverse Young Adults: Survey Study. *JMIR Formative Research*, *7*. <https://doi.org/10.2196/45952> |
| 95 | Caldarera, A. M., Vitiello, B., Bechis, D., & Baietto, C. (2023). Promoting sexual health in transgender and gender diverse adolescents through an online sexuality psychoeducation program for parents: A case study. *Clinical Child Psychology and Psychiatry*, *28*(3), 1038–1052. <https://doi.org/10.1177/13591045231160641> |
| 96 | Chen, D., Shen, E., Kolbuck, V. D., Sajwani, A., Finlayson, C., & Gordon, E. J. (2024). Co-design and usability of an interactive web-based fertility decision aid for transgender youth and young adults. *Journal of Pediatric Psychology*. <https://doi.org/10.1093/jpepsy/jsae032> |
| 97 | Coulter, R. W. S., Dougherty, M., Clark, M. B., Wilson, A., Miller, E., & Ragavan, M. I. (2024). Online Human-Centered Design Methods are Acceptable, Appropriate, and Feasible for Generating Adolescent Relationship Abuse Intervention Ideas With Sexual and Gender Diverse Youth. *Journal of Adolescent Health*, *75*(4), 656–664. <https://doi.org/10.1016/j.jadohealth.2024.06.001> |
| 98 | Cronesberry, K., & Ward, L. (2024). Exploring gender diverse young adults’ gender identity development in online LGBTQIA + communities. *International Journal of Transgender Health*. <https://doi.org/10.1080/26895269.2024.2344534> |
| 99 | DeGuzman, P. B., Lyons, G. R., Azar, F. N., Kimble, A., Huang, G., Rheuban, K., & Gray, S. H. (2024). Impact of Telemedicine on Access to Care for Rural Transgender and Gender-Diverse Youth. *Journal of Pediatrics*, *267*. <https://doi.org/10.1016/j.jpeds.2024.113911> |
| 100 | Delmonaco, D., & Haimson, O. L. (2023). “Nothing that I was specifically looking for”: LGBTQ + youth and intentional sexual health information seeking. *Journal of LGBT Youth*, *20*(4), 818–835. <https://doi.org/10.1080/19361653.2022.2077883> |
| 101 | Delmonaco, D., Li, S., Paneda, C., Popoff, E., Hughson, L., Jadwin-Cakmak, L., Alferio, J., Stephenson, C., Henry, A., Powdhar, K., Gierlinger, I., Harper, G. W., & Haimson, O. L. (2023). Community-Engaged Participatory Methods to Address Lesbian, Gay, Bisexual, Transgender, Queer, and Questioning Young People’s Health Information Needs With a Resource Website: Participatory Design and Development Study. *JMIR Formative Research*, *7*(1). <https://doi.org/10.2196/41682> |
| 102 | Dos Santos, F., Garofalo, R., Kuhns, L., Wood, O., Scherr, T., & Schnall, R. (2024). Usability of a Mobile Health Technology for HIV Testing among Sexual Minorities. *Studies in Health Technology and Informatics*, *315*, 744–745. <https://doi.org/10.3233/SHTI240309> |
| 103 | Erenrich, R. K., Braun, R. A., Torres-Mendoza, D. M., Stevenson, O. L., Doan, T.-H. P., & Klausner, J. D. (2024). *Effectiveness of PrEPTECH: Findings From a 180-Day Randomized Controlled Trial of a Pre-Exposure Prophylaxis Telehealth Intervention*. [www.jaids.com](http://www.jaids.com) |
| 104 | Gamarel, K. E., Darbes, L. A., Wall, K. M., Jones, J., Washington, C., Rosso, M., Felder Claude, K., Hightow-Weidman, L. B., Sullivan, P. S., & Stephenson, R. (2023). A Relationship-Focused HIV Prevention Intervention for Young Sexual Minority Men in the United States: A Pilot Randomized Controlled Trial of the We Prevent Intervention. *AIDS and Behavior*, *27*(8), 2703–2719. <https://doi.org/10.1007/s10461-023-03994-5> |
| 105 | Gamson, J., & Hertz, R. (2023). “But Everything Else, I Learned Online”: School-Based and Internet-Based Sexual Learning Experiences of Heterosexual and LGBQ + Youth. *Qualitative Sociology*, *46*(4), 461–485. <https://doi.org/10.1007/s11133-023-09550-w> |
| 106 | Haggipavlou, L., & Hamshaw, R. J. T. (2023). Barriers to PrEP Uptake in Young U.K. Men Who Have Sex With Men. *Journal of Prevention and Health Promotion*, *4*(3–4), 404–433. <https://doi.org/10.1177/26320770231199419> |
| 107 | Halloran, J., Smidt, A. M., Morrison, A., Cron, J., Kallen, A. N., & Olezeski, C. L. (2023). Reproductive and Fertility Knowledge and Attitudes Among Transgender and Gender-Expansive Youth: A Replication and Extension. *Transgender Health*, *8*(4), 328–336. <https://doi.org/10.1089/trgh.2021.0080> |
| 108 | Herry, E., Rodan, S. M., Martin, M., Sanjak, M. M., & Mulvey, K. L. (2024). White American transgender adults’ retrospective reports on the social and contextual aspects of their gender identity development. *British Journal of Developmental Psychology*. https://doi.org/10.1111/bjdp.12480 |
| 109 | Hiebert, A., & Kortes-Miller, K. (2023). Finding home in online community: exploring TikTok as a support for gender and sexual minority youth throughout COVID-19. *Journal of LGBT Youth*, *20*(4), 800–817. <https://doi.org/10.1080/19361653.2021.2009953> |
| 110 | Hobaica, S., Szkody, E., Sotomayor, I., Liao, J., & Schleider, J. L. (2024). Sexual Health Education Experiences and Recommendations from the Perspective of LGBTQ+ Youth. *Journal of Sex Research*. <https://doi.org/10.1080/00224499.2024.2355564> |
| 111 | Horvath, K. J., Helm, J. L., Black, A., Chase, G. E., Ma, J., Klaphake, J., Garcia-Myers, K., Anderson, P. L., & Baker, J. V. (2024). A Pilot Randomized Controlled Trial of an mHealth Intervention to Improve PrEP Adherence Among Young Sexual Minority Men. *AIDS and Behavior*, *28*(8), 2804–2820. <https://doi.org/10.1007/s10461-024-04374-3> |
| 112 | Horvath, K. J., Ma, J., Storholm, E. D., Black, A., Klaphake, J., & Baker, J. V. (2023). The Use of Daily and On-Demand Oral Pre-Exposure Prophylaxis Dosing Strategies Among Young Adult Gay, Bisexual and Other Men who have Sex with Men Enrolled in an mHealth Adherence Intervention. *AIDS and Behavior*, *27*(11), 3632–3644. <https://doi.org/10.1007/s10461-023-04077-1> |
| 113 | Huebner, D. M., Barnett, A. P., Baucom, B. R. W., & Guilamo-Ramos, V. (2023). Effects of a Parent-Focused HIV Prevention Intervention for Young Men Who have Sex with Men: A Pilot Randomized Clinical Trial. *AIDS and Behavior*, *27*(5), 1502–1513. <https://doi.org/10.1007/s10461-022-03885-1> |
| 114 | Inwards-Breland, D. J., Yeh, D., Marinkovic, M., Richardson, T. R., Marino-Kibbee, B., Bayley, A., & Rhee, K. E. (2024). Facilitators and barriers to using telemedicine for gender-affirming care in gender-diverse youth: A qualitative study. *Journal of Telemedicine and Telecare*. <https://doi.org/10.1177/1357633X241231015> |
| 115 | Jayne, P. E., Szucs, L. E., Lesesne, C. A., Grose, R. G., & Johns, M. M. (2024). “I wouldn’t have felt so alone”: The sexual health education experiences of transgender and gender diverse youth living in the southeastern United States. *Perspectives on Sexual and Reproductive Health*, *56*(2), 158–170. <https://doi.org/10.1111/psrh.12258> |
| 116 | Kahn, N. F., Asante, P. G., Guler, J., Reyes, V., Anan, Y., Bocek, K., Kidd, K. M., Richardson, L. P., Christakis, D. A., Pratt, W., & Sequeira, G. M. (2024). Caregiver Perspectives on Receiving Gender-Affirming Care with Their Transgender and Gender Diverse Adolescents via Telemedicine. *LGBTQ+ Family: An Interdisciplinary Journal*, *20*(3), 190–200. <https://doi.org/10.1080/27703371.2024.2317139> |
| 117 | Katz-Wise, S. L., Godwin, E. G., Medzhitova, Y., Moore, L. B. M., Parsa, N., Hill, A., Oparah, N., Bogart, L. M., Rosal, M. C., Pullen Sansfaçon, A., Ehrensaft, D., Nishman, M. M., & Austin, S. B. (2024). Development of a family-level intervention for families with transgender and/or nonbinary youth: Lessons and recommendations. *Journal of Family Psychology*. <https://doi.org/10.1037/fam0001262> |
| 118 | Kaufman, M. R., Wright, K., Eschliman, E. L., Levine, D., & Simon, J. (2023). A Mobile App to Assist the Mentors of African American Young Men Who Have Sex With Men: Usability Study. *JMIR Formative Research*, *7*(1). <https://doi.org/10.2196/48515> |
| 119 | Maragh-Bass, A. C., Williams, T., Agarwal, H., Dulin, A. K., Sales, J., Mayer, K. H., & Siegler, A. J. (2023). Exploring Stigma, Resilience, and Alternative HIV Preventive Service Delivery Among Young Men who Have Sex with Men of Color. *Clinical Nursing Research*, *32*(7), 1046–1056. <https://doi.org/10.1177/10547738231184295> |
| 120 | Muessig, K. E., Vecchio, A. C., Hanshaw, B. D., Soberano, Z., Knudtson, K. A., Claude, K. F., Larsen, M. A., & Hightow-Weidman, L. B. (2024). Barriers, Facilitators and Opportunities for HIV Status Disclosure Among Young Men Who Have Sex With Men: Qualitative Findings from the Tough Talks Intervention. *AIDS and Behavior*. <https://doi.org/10.1007/s10461-024-04406-y> |
| 121 | Ocasio, M. A., Fernandez, M. I., Cortese, S., & Kampa, K. (2023). Piloting a digital campaign to promote awareness of the Louisiana TelePrEP program among sexual and gender minority young adults. *PLoS ONE*, *18*(8 August). <https://doi.org/10.1371/journal.pone.0290149> |
| 122 | Owens, C. (2024). Attitudes Toward Using and Willingness to Use a Hypothetical Online Sex Communication Intervention for Parents of Sexual and Gender Minority Adolescents. *Archives of Sexual Behavior*, *53*(8), 3119–3127. <https://doi.org/10.1007/s10508-024-02946-x> |
| 123 | Penfold, A., Callaghan, P., & Urry, K. (2024). Online Communities and Identity: Experiences of LGBTQIA+ Emerging Adults Engaging With LGBTQIA+ Online Content During the COVID-19 Pandemic. *Psychology of Popular Media*. <https://doi.org/10.1037/ppm0000529> |
| 124 | Plant, A., Sparks, P., Creech, D. N., Morgan, T., Klausner, J. D., Rietmeijer, C., & Montoya, J. A. (2024). Developing an mHealth program to improve HIV care continuum outcomes among young Black gay and bisexual men. *BMC Public Health*, *24*(1). <https://doi.org/10.1186/s12889-024-18652-1> |
| 125 | Reiter, P. L., Gower, A. L., Kiss, D. E., Shoben, A. B., Katz, M. L., Bauermeister, J. A., Paskett, E. D., & McRee, A. L. (2023). Efficacy of the Outsmart HPV Intervention: A Randomized Controlled Trial to Increase HPV Vaccination among Young Gay, Bisexual, and Other Men Who Have Sex with Men. *Cancer Epidemiology Biomarkers and Prevention*, *32*(6), 760–767. <https://doi.org/10.1158/1055-9965.EPI-23-0007> |
| 126 | Roden, R. C., Billman, M. G., Mullin, R., Francesco, A., & Essayli, J. H. (2023). Rural Location of Residence is Not Associated With Use of Telemedicine for Initial Medical Contact for Gender-Related Healthcare. *Journal of Adolescent Health*, *73*(5), 940–945. <https://doi.org/10.1016/j.jadohealth.2023.06.027> |
| 127 | Sethness, J. L., Sequeira, G. M., Kidd, K. M., Evans, Y. N., Lin, Y. H., Pratt, W., Christakis, D., Richardson, L. P., & Kahn, N. F. (2024). Guardian Reasons for Accessing Their Transgender and Gender-Diverse Adolescent’s Patient Portal Account. *Journal of Adolescent Health*, *75*(3), 516–518. <https://doi.org/10.1016/j.jadohealth.2024.06.002> |
| 128 | Shah, T. N., Parodi, K. B., Holt, M. K., Green, J. G., Katz-Wise, S. L., Kraus, A. D., Kim, G. S., & Ji, Y. (2024). A Qualitative Exploration of How Transgender and Non-binary Adolescents Define and Identify Supports. *Journal of Adolescent Research*, *39*(1), 133–164. <https://doi.org/10.1177/07435584221123123> |
| 129 | Sinno, J., Macapagal, K., & Mustanski, B. (2024). Social Media and Online Dating Safety Practices by Adolescent Sexual and Gender Diverse Men: Mixed-Methods Findings From the SMART Study. *Journal of Adolescent Health*, *74*(1), 113–122. <https://doi.org/10.1016/j.jadohealth.2023.07.030> |
| 130 | Swendeman, D., Rotheram-Borus, M. J., Arnold, E. M., Fernández, M. I., Comulada, W. S., Lee, S. J., Ocasio, M. A., Ishimoto, K., Gertsch, W., Duan, N., Reback, C. J., Murphy, D. A., Lewis, K. A., Abdalian, S. E., Bolan, R., Bryson, Y., Cortado, R., Flynn, R., Kerin, T., … Weiss, R. E. (2024). Optimal strategies to improve uptake of and adherence to HIV prevention among young people at risk for HIV acquisition in the USA (ATN 149): a randomised, controlled, factorial trial. *The Lancet Digital Health*, *6*(3), e187–e200. <https://doi.org/10.1016/S2589-7500(23)00252-2> |
| 131 | Wongsomboon, V., Queiroz, A. A. F. L. N., Alvarado Avila, A., Mongrella, M., Saber, R., Li, D. H., Moskowitz, D. A., Mustanski, B., & Macapagal, K. (2023). Acceptability of ‘Humpr’: An Online Tool to Educate Adolescent Sexual Minority Males About Sexual Networking Applications. *Journal of Sex Research*. <https://doi.org/10.1080/00224499.2023.2273935> |
| 132 | Zapata, J. P., Queiroz, A., Rodriguez-Diaz, C. E., & Mustanski, B. (2024). Factors Associated with HIV Testing Among Spanish and English-Speaking Latino Adolescents Aged 13–18. *AIDS and Behavior*, *28*(1), 343–356. <https://doi.org/10.1007/s10461-023-04206-w> |
