# Supplementary material for: Mapping the missing: a scoping review identifying critically underrepresented LGBTQI+ youth within online sexual, reproductive, and transgender healthcare research
Source: Sex Reprod Health Matters. 2026 May 29;33(1):2679359. doi: 10.1080/26410397.2026.2679359 (PMC13288906; doi:10.1080/26410397.2026.2679359)
Supplement: Supplementary File 6. Data analysis [file ZRHM_A_2679359_SM1066.docx]

### Supplementary File 6. Data analysis

| **Review question** | **Variable** | **Data categorisation** |
| --- | --- | --- |
| RQ1 | Area of health | Papers were deductively categorised as belonging to sexual, reproductive, or transgender health. This was identified from the health topic of the paper from the title, abstract, introduction, and methods, for example, a paper about HIV testing was categorised as sexual health, a paper about pregnancy prevention was categorised as reproductive health, and a paper about gender identity was categorised as transgender health. |
|  | Health topic | Papers were inductively categorised as focusing on one or more health topic within the areas of sexual health, reproductive health, and transgender health, such as HIV prevention; STI prevention; HIV and STI prevention; HIV management; HIV stigma reduction; Pregnancy prevention; Sexual health (per se); reproductive health (per se); gender identity/expression; and gender affirmation/transition. This was identified from the title, aim, and/or methods. |
|  | Healthcare types | Papers were deductively categorised as focusing on one or more of Education/Information, Non-clinical Support, and Clinical Care, identified from the title, aim, and/or methods (intervention/service description). *Education/Information* refers to services and interventions that impart information only typically to achieve an increase in knowledge (e.g., information about STIs/HIV; contraception; or gender expression and transition). *Non-clinical Support* refers to services and interventions that provide non-clinical emotional or practical support, beyond information, typically to achieve a desired outcome (e.g., peer communication for HIV stigma reduction; reminders for increased PrEP adherence; or skill building for increased skills for partner notification). *Clinical Care* refers to services and interventions for medical care, specific to testing, diagnosing, treating, and managing sexual and reproductive health issues or gender affirming care (e.g., STI/HIV testing; uptake or maintenance of PrEP; consultations for gender affirming hormones). |
|  | Online platforms/ types | Papers were inductively categorised as focussing on one or more type of online platform, using verbatim terms extracted from the title, abstract, aim, and/or methods (service/intervention description). |
| RQ2 | Target LGBTQI+ population | Data were deductively categorised into one of 8 categories: LGB/Sexual minority; LGBT+/Sexual and gender minority; GBMSM (cisgender, gender inclusive AMAB, or not specified); WSW/WSWM/Sexual minority women (cisgender, gender inclusive AFAB, or not specified); Trans and gender diverse; Trans men; Trans women. |
|  | PROGRESS-Plus | Papers were deductively categorised as considering Place of Residence, Race/Ethnicity, Occupation, Gender/Sex, Religion, Education, Socio-Economic Status, Social Networks (PROGRESS), Features of relationships, Time-dependent relationships, Sexual identity/orientation, Age, Disability, (Plus) (Kavanagh & Oliver, 2008; O’Neill et al., 2014) and Living with HIV in recruitment. |
| RQ3 | How theory/ model/ framework was used | Papers were deductively categorised as having used theory in a manner that is either ‘Applied’ or ‘Descriptive’, identified from the introduction, methods, analysis, and/or discussion. Applied refers to the use of theory/models/frameworks in a practical, hands-on manner to directly guide intervention development, data analysis, or strategy formulation. These frameworks are typically used to drive concrete action and outcomes, addressing real-world problems. Descriptive refers to when frameworks are used to contextualise the study or provide insight into the broader social or structural dynamics that affect the research topic. These frameworks help set the stage for understanding the study or its findings within a wider societal, cultural, or political context, often illuminating issues like inequality, identity, and power. Papers could also be categorised as ‘Unclear’ if the manner in which the framework was used was not clearly reported. |
